# Impaired adult neurogenesis is an early event in Alzheimer’s disease neurodegeneration, mediated by intracellular Aβ oligomers

**DOI:** 10.1038/s41418-019-0409-3

**Published:** 2019-10-07

**Authors:** Chiara Scopa, Francesco Marrocco, Valentina Latina, Federica Ruggeri, Valerio Corvaglia, Federico La Regina, Martine Ammassari-Teule, Silvia Middei, Giuseppina Amadoro, Giovanni Meli, Raffaella Scardigli, Antonino Cattaneo

**Affiliations:** 1 Department of Science, University “Roma Tre”, Roma, Italy; 2 European Brain Research Institute (EBRI), Roma, Italy; 3 Consiglio Nazionale delle Ricerche (CNR), Institute of Cell Biology and Neurobiology, Roma, Italy; 4 Consiglio Nazionale delle Ricerche (CNR), Institute of Translational Pharmacology, Roma, Italy; 5 Scuola Normale Superiore, Pisa, Italy; 6 IRCSS Fondazione Santa Lucia, Roma, Italy

**Keywords:** Cell biology, Neurological disorders

## Abstract

Alterations of adult neurogenesis have been reported in several Alzheimer's disease (AD) animal models and human brains, while defects in this process at presymptomatic/early stages of AD have not been explored yet. To address this, we investigated potential neurogenesis defects in Tg2576 transgenic mice at 1.5 months of age, a prodromal asymptomatic age in terms of Aβ accumulation and neurodegeneration. We observe that Tg2576 resident and SVZ-derived adult neural stem cells (aNSCs) proliferate significantly less. Further, they fail to terminally differentiate into mature neurons due to pathological, tau-mediated, and microtubule hyperstabilization. Olfactory bulb neurogenesis is also strongly reduced, confirming the neurogenic defect in vivo. We find that this phenotype depends on the formation and accumulation of intracellular A-beta oligomers (AβOs) in aNSCs. Indeed, impaired neurogenesis of Tg2576 progenitors is remarkably rescued both in vitro and in vivo by the expression of a conformation-specific anti-AβOs intrabody (scFvA13-KDEL), which selectively interferes with the intracellular generation of AβOs in the endoplasmic reticulum (ER). Altogether, our results demonstrate that SVZ neurogenesis is impaired already at a presymptomatic stage of AD and is caused by endogenously generated intracellular AβOs in the ER of aNSCs. From a translational point of view, impaired SVZ neurogenesis may represent a novel biomarker for AD early diagnosis, in association to other biomarkers. Further, this study validates intracellular Aβ oligomers as a promising therapeutic target and prospects anti-AβOs scFvA13-KDEL intrabody as an effective tool for AD treatment.

## Introduction

Neurogenesis persists in two areas of the adult mammalian brain, the subventricular zone (SVZ) and the hippocampal dentate gyrus (DG). The extent and relevance of adult neurogenesis (AN) in humans is currently debated [1–6]. However, even in the case of low neurogenesis rates under homeostatic conditions in the human brain, the possibility to induce human neural precursor to generate new neurons is an attractive prospect for neuroreplacement therapy in neurodegenerative diseases, such as Alzheimer’s disease (AD). Remarkably, it has been recently described that neurogenesis persists in cognitively healthy people until the end of life, but drops off dramatically as AD pathology takes hold [7]. Previous studies also reported that the expression of neurogenesis markers is decreased in SVZ [8] and DG regions [9] of postmortem human AD brains. Thus, AN alteration could be proposed as a biomarker of AD progression in humans, but it is not known whether such changes are an early event or a late consequence of the disease. Since a systematic study of dynamic alterations of AN in humans is hampered by the lack of tunable approaches and by the complexity of AD pathology in the human population, animal models are necessary for mechanistic studies of AN. Different transgenic AD animal models show an altered neurogenesis in both SVZ and DG neurogenic niches, with a majority of studies reporting reduced neurogenesis and some others observing, instead, an increased generation of new neurons [10–13]. These discrepancies might be due, in part, to the different timing of the observed AN alterations, with respect to the progression of neurodegeneration. According to the different hypotheses formulated about the role of neurogenesis in AD, enhanced AN might occur in diseased brain as a homeostatic self-repair mechanism [14]; alternatively, decreased neurogenesis might contribute to the onset of neurodegeneration [15, 16]. In this view, AD-causing molecules, such as the Amyloid-beta (Aβ) peptide, would deregulate AN, facilitating disease progression [17]. However, it is still unclear whether and how the pathophysiological environment in the AD brain, and in particular the different Aβ species, affects AN. A number of studies have addressed this question by investigating how diverse Aβ peptides modulate adult neural stem cells (aNSCs) biology. The results are controversial, reporting that extracellularly administered Aβ either decreases [18, 19] or increases [20–22] NSCs proliferation and thus neurogenesis. These discrepancies are likely due to the different Aβ species used, since discrimination among different Aβ assembly states or conformations was not always investigated [21, 23]. Moreover, no previous study explored the role in AN of intracellular Aβ generation and oligomerization, one of the earliest events in AD pathogenesis [24, 25].

In this work we investigated whether alterations in AN represent an early event in AD neurodegeneration and whether the intracellular generation and oligomerization of endogenous Aβ plays a causal role in these alterations. We analyzed the proliferative and differentiative features of resident and SVZ-derived aNSCs in Tg2576 transgenic mice, a well-characterized animal model of AD and Aβ accumulation [26], at a very early presymptomatic age. Moreover, we investigated if intracellularly formed Aβ oligomers (AβOs) in Tg2576 modulate aNSCs biology in these mice, and provided a proof-of-concept for an innovative disease modifying approach based on intrabody conformational selective and subcellularly localized gene therapy.

## Materials and methods

### Study design

This study was designed to determine whether AN alteration represents an early event in AD neurodegeneration. We analyzed the proliferative and differentiative features of resident and SVZ-derived adult neural stem cells in the APP line Tg2576, which expresses the human APP with the Swedish mutation (APPKM670/671NL), directed by the hamster prion promoter [26], and represent a well-characterized animal model of AD and Aβ accumulation. We examined the cell biology of neural stem cells of the SVZ of young mice (1.5-month-old), both in vivo and in vitro. For in vivo studies in mice, we used sample sizes between four and six to test significant differences. For proliferation and differentiation studies in vitro we used minimal replicates of three for each experimental condition. For AβOs intracellular targeting we used a subcellularly localized conformational-selective interference (CSI) approach, based on the lentiviral-mediated expression of a recombinant antibody fragment against AβOs in the endoplasmic reticulum (ER), namely scFvA13-KDEL [24].

### Experimental animals

Tg2576 and wild-type littermates were used at 1.5 months of age. All experiments with transgenic and control mice were conducted according to national and international laws for laboratory animal welfare and experimentation (EEC council directive 86/609, OJ L 358, 12 December 1987; Dlgs 116/92). In detail, mice were grouped in standard cages (hardwoods bedding) in conventional animal facility (12 h light/dark cycle). Groups included four mice per cage, balanced for genotype and mice were monitored for health and welfare for the whole duration of the experiments. Only mice without stress or discomfort signs (including hair loss, stereotyped behaviors) and weight ranging between 20–30 g were included in the study.

### Brain dissection and tissue processing

Prior to brain dissection, adult mice were anesthetized by intraperitoneal injections with about 1 ml of 2.5% 2,2,2-tribromethanol (Sigma-Aldrich) and intracardially perfused with 4% paraformaldehyde (PFA). The whole brain was therefore extracted and the fixation continued in 4% PFA overnight at 4 °C. After cryoprotection in 30% sucrose, brains were cryo-sectioned at 40 μm of thickness, and slices encompassing the SVZ and the olfactory bulbs (OB) were analyzed by immunohistochemistry.

### Neural stem cell cultures

NSCs cultures were performed as described in Scardigli et al. [13]. In detail, 1.5-month-old mice (wild type or Tg2576) were anesthetized as described before and killed by decapitation. Brains were extracted out of the skull and then located in a brain slicer (Zivic Instrument) in order to obtain coronal slices of 1 mm of thickness. SVZ regions were dissected out of the brain slices by microdissection performed under a stereomicroscope, and cells were isolated by enzymatic digestion (1.33 mg/ml trypsin, 0.7 mg/ml hyaluronidase, and 0.2 mg/ml kynurenic acid) (Sigma-Aldrich) for 30 min at 37 °C and mechanical dissociation with small-bore Pasteur pipette. Cells were plated at 10^4^ cells/cm^2^ cells density and cultured in growth medium, consisting in Dulbecco’s modified Eagle’s medium (DMEM)/F12 medium supplemented with B27 (Invitrogen, San Diego, CA, http://www.invitrogen.com), epidermal growth factor (EGF), and basic fibroblast growth factor (bFGF), (20 and 10 ng/ml, respectively; Peprotech, Rocky Hill, NJ, http://www.peprotech.com) (growing medium) in a humidified incubator at 37 °C in 5% CO_2_ for 3 weeks. Growth factors were replenished weekly.

For primary neurospheres quantification, neurospheres were counted after 1 week of culture and their size was measured. For neurospheres quantification in number we took light microscope images (five fields for each sample) at lower magnification (×10) and we counted neurospheres manually. The size of neurospheres was expressed as their diameter in phase contrast pictures. Brightness and contrast images of live neurospheres were taken at a Nikon Eclipse Inverted TE 2000-E microscope, using NIS Elements 3.0 software.

For neurospheres culture propagation, primary neurospheres (50–100 μm in diameter) were subcultured by mechanical dissociation into single cells. This procedure was repeated on newly formed neurospheres. Growing medium was half replaced every other day.

To assess for differentiation, neurospheres were dissociated into single cells and transferred onto matrigel-coated glass coverslips (12 mm diameter) in differentiating medium (growth medium without EGF and FGF) at 5 × 10^4^ cells/cm^2^. Five days after plating, cultures were fixed in 4% PFA at RT for 10 min and processed for immunocytochemistry.

### Analysis of cell proliferation

5 × 10^3^ viable cells (2.5 × 10^3^/cm^2^ cell density) were initially plated in a T24 multiwell in growing medium. After 7 days in vitro (DIV 7), the total number of viable cells was counted by Trypan blue exclusion, and all cells were replated under the same cell density. This procedure was repeated for at least four subculture passages, in order to provide the statistical mean of the proliferation index of Tg2576 vs wild-type progenitors. Cell proliferation between DIV 0 and DIV 14 was either expressed as Fold Increase in cell number (F.I.), calculated according to the following formula: (n° of cells at DIV 14 − n° of cells at DIV 0)/the initial number of seeded cells.

### In Vivo and In Vitro bromodeoxyuridine labeling

For in vivo labeling, bromodeoxyuridine (BrdU; Sigma-Aldrich) was administered to Tg2576 and control adult mice (1.5-month-old) at 100 mg/kg by daily intraperitoneal injections for 5 days. Animals were then sacrificed either 2 (for SVZ) or 21 days (for OB) after the last injection, and brains were collected and processed as described before.

For in vitro BrdU labeling neurospheres were dissociated into single cells and transferred onto matrigel-coated glass coverslips (12 mm diameter) in growth medium. The day after 20 µM BrdU was administered to cell cultures for 30 min or 2 h depending on the experiments. Treated cells were fixed 24 h later and processed for immunohistochemistry for anti BrdU staining (see Table I).

### Immunocytochemistry on brain sections and neurospheres

Immunohistochemistry of SVZ and OB was performed on 40-μm serial free-floating sections. To improve the efficiency of BrdU detection, sections and cells, prior to antibody staining, were exposed to 2 N HCl for 30 min (for sections) or 15 min (for cells), respectively, at 37 °C and then washed with 0.1 M sodium borate buffer pH 8.5 for 10 min (to allow the denaturation of DNA, necessary to expose the BrdU). Upon fixation, sections or cells were permeabilized in blocking solution (0.3 or 0.1% Triton X-100, respectively, in PBS, 3 or 10% normal donkey serum and normal goat serum, respectively) and then incubated with the antibody of interests (Table I). The total number of cells in each field was determined by counterstaining cell nuclei with 4,6-diamidine-2-phenylindole dihydrochloride (DAPI; Sigma-Aldrich; 50 mg/ml in PBS for 15 min at RT). Immunostained sections and cells were mounted in Aqua-Poly/Mount (Polysciences) and analyzed at epi-fluorescent or confocal microscopy, using a Nikon Eclipse 90i microscope (Nikon) or a TCS SP5 microscope (Leica Microsystem). Z-stacks images were captured at 1 μm intervals with a ×40 or ×63 objectives and a pinhole of 1.0 Airy unit. Analyses were performed in sequential scanning mode to rule out cross-bleeding between channels. Fluorescence intensity quantification was performed with ImageJ software.

### Western blot assay for Aβ

Samples (conditioned media, CM, or cell lysates) were diluted in NuPAGE™ LDS sample buffer (ThermoFisher Scientific, NP0007) and 10% DTT solution 1 M (Applichem, Germany, A3668, 0050), boiled 10 min, loaded in precasted NuPAGE™ Novex™ 10% Bis-Tris Midi Protein Gels (ThermoFisher Scientific, WG1201A), running in NuPAGE MES SDS running buffer (ThermoFisher Scientific, NP0002). The semidry blot was then done onto nitrocellulose membrane filters, 0.22 μm (GE Healthcare, 10600001). The membrane filter was boiled in PBS to increase the detection of low molecular weight (MW) bands. After the incubation with the blocking solution (TBS 0.05% tween, 5% dry milk), primary antibodies were used in TBS 0.05% tween, 2.5% dry milk at the concentration reported in Table I. Following the incubation with secondary peroxidase-coupled anti-mouse or anti-rabbit antibodies, ECL (GE Healthcare, RPN2209) chemiluminescent detection was performed. Quantitative densitometric analysis was performed using ImageJ software (http://imagej.nih.gov/ij/), according to the procedure described in the dedicated section.

### Dot blot analysis

CM and cellular samples were spotted onto nitrocellulose membrane filters 0.22 μm (GE Healthcare, 10600001). After incubation with the blocking solution (TBS 0.05% tween, 10% dry milk or  TBS 0.01% tween, 10% dry milk for anti-oligomer pAbA11) primary antibodies were used in TBS 0.05% tween, 5% dry milk or TBS 0.01% tween, 5% dry milk (for anti-oligomer pAbA11). Purified recombinant anti-AβOs scFvA13 (3.5 μg/ml) was used as described [24] and immunodetection was performed by anti-V5 tag (Fig. 2e) or by anti-His tag (Fig. 4c), which respectively recognizes the V5 tag or C-terminal 6xHis tag of recombinant scFvA13. After incubation with secondary peroxidase coupled anti-mouse or anti-rabbit antibodies, ECL (GE Healthcare, RPN2209) chemiluminescent detection was performed. Serial dilution curves of cellular samples were preliminarily tested to obtain nonsaturating condition of immunodetection and samples were loaded at 1 μg/spot, whereas 1 μl of CM were spotted. For each specific antibody staining protein loading was normalized to the corresponding Ponceau staining. Blots were scanned and quantitative densitometric analysis was performed by using ImageJ software (http://imagej.nih.gov/ij/), as described in Supplementary Materials and Methods.

### Statistical analysis

#### Animal studies

Power analysis was conducted to estimate the appropriate sample size by setting the probability of a Type I error (α) at 0.05, power at 0.95, and effect size at 0.4. To minimize the effects of subjective bias we used randomization procedures for allocating animals to experimental groups and treatments and blind analysis of results. The statistical analyses were conducted using Mann–Whitney test.

#### In vitro studies

Statistical analyses were conducted using either Mann–Whitney test or by unpaired Student’s *t* test, as specified in each figure legend. Statistical analysis in Fig. 3b and Supplementary Fig. S6 was performed by one-way ANOVA test.

Error bars on graphs indicate ± SEM. Significance markers on figures are from post hoc analyses (n.s., not significant; *P *<* 0.05, **P *<* 0.01, ***P *<* 0.001).

## Results

### Olfactory bulb neurogenesis is impaired in presymptomatic Tg2576 mice

**Fig. 1:**
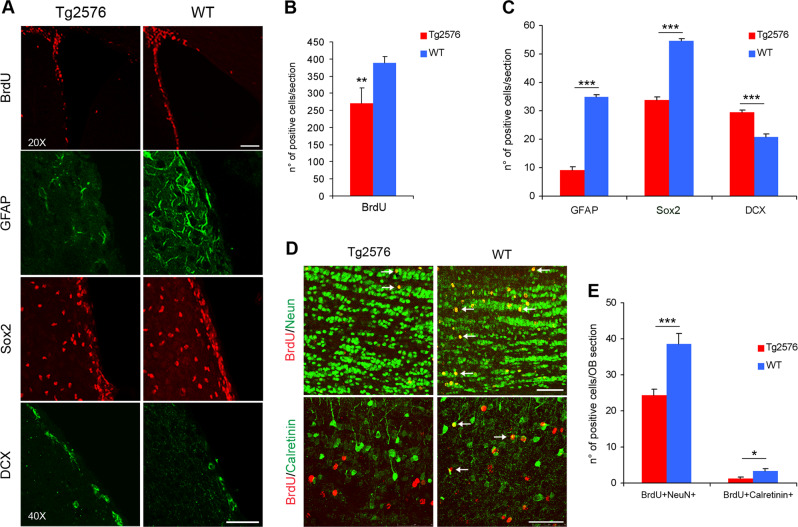
In vivo SVZ and OB neurogenesis is affected in Tg2576 mice. **a** Immunofluorescence staining for BrdU, progenitors, and neuroblasts markers in adult SVZ of Tg2576 and wild type (WT) mice. Immunostaining for incorporated BrdU in progenitor cells shows reduced proliferation in Tg2576 SVZ niche (red signal in top panels), while there is a significant reduction of aNSCs compartment (GFAP^+^ and Sox2^+^ cells) and an increase in DCX^+^ neuroblasts. Scale bar 100 µm, ×20 magnification (for BrdU) and 50 µm, ×40 magnification, zoom 1.3 (for SVZ markers). The histograms represent the quantification of BrdU (**b**) or Sox2, GFAP, and DCX positive cells (**c**) in Tg2576 (red) and WT (blue) SVZ. Data are means  ± SEM of five individual animals (*n* = 5) for each experimental group. **P < 0.01, ***P < 0.001, significantly different from WT, Mann–Whitney test. **d** Immunostaining for BrdU and NeuN (red and green, respectively, top panels, ×40 magnification) and BrdU and Calretinin (red and green, respectively, bottom panels, ×63 magnification) shows a significant reduction of newborn Calretinin interneurons in Tg2576 olfactory bulbs compared with WT. Scale bar 50 µm. **e** Quantification of double positive cells in Tg2576 (red) and WT (blue). Data are means  ± SEM of five individual animals (*n* = 5) for each experimental group. *P < 0.05, ***P < 0.001, significantly different from WT, Mann–Whitney test

To identify alterations of AN at early prodromal stages of AD, we measured the proliferative rate of SVZ NSCs by in vivo BrdU labeling in 1.5-month-old APP Tg2576 mice, which represent a slow progressive AD model developing amyloid-beta (Aβ) plaques around 9–13 months of age [26, 27], with the earliest synaptic and learning deficits observed at 3 months of age [28]. Anti-BrdU staining of brain sections encompassing the entire SVZ, revealed fewer BrdU positive cells in Tg2576 SVZ, compared with control wild type (WT) mice (Fig. 1a, upper panels, and b: number of positive cells/section: Tg2576 271 ± 4; WT 389 ± 2, *P* *<* 0.01), indicating a reduced proliferation of resident Tg2576 SVZ progenitors. While in WT animals SVZ proliferating cells are mainly Sox2^+^ and GFAP^+^ progenitors, in Tg2576 mice the BrdU^+^ cells are equally distributed between progenitors and neuroblasts (Supplementary Fig. S1A). The expression of progenitors and neuroblasts markers, such as Sox2, GFAP, and Doublecortin (DCX), was analyzed by immunofluorescence: Fig. 1a shows that in Tg2576 SVZ there is a significant reduction in the number of Sox2 and GFAP positive cells, while DCX^+^ neuroblasts are more represented, compared with WT (Fig. 1c). Consistently, OB neurogenesis was also affected (Fig. 1d, e), with a significant decrease of newborn neurons (n° of NeuN^+^BrdU^+^ cells/OB section: Tg2576 24.28 ± 7.57; WT 38.56 ± 12.27; *P* < 0.001). In particular, we found less Calretinin positive interneurons in Tg2576 OB (n° of Calretinin^+^BrdU^+^ cells/OB section: Tg2576 1.3 ± 0.15; WT 3.3 ± 0.19; *P* *<* 0.05). Altogether, these results demonstrated that SVZ neurogenesis is impaired in presymptomatic Tg2576 mice.

### In vitro proliferation and differentiation defects of Tg2576 neural stem cells correlate with Aβ/AβOs levels

**Fig. 2:**
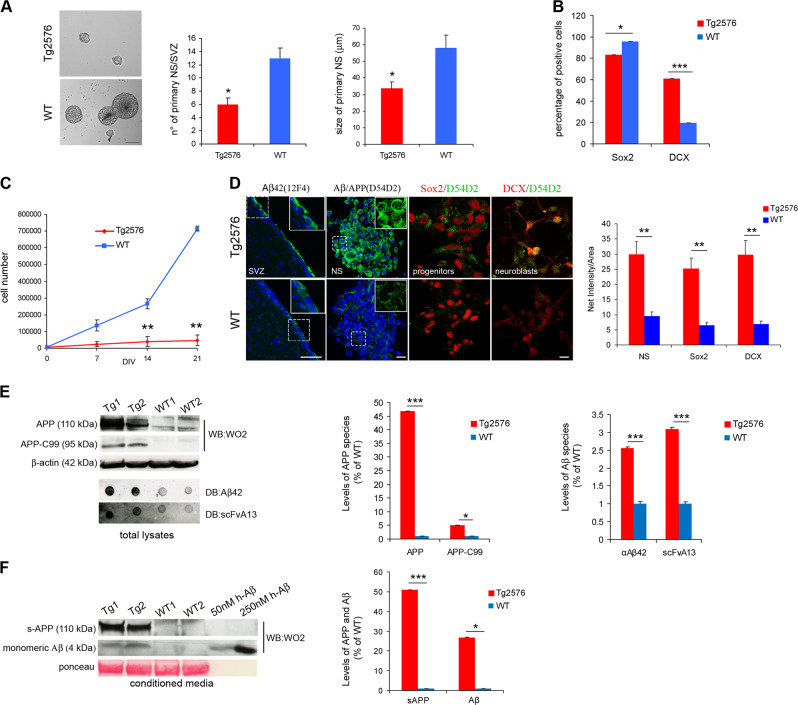
Proliferative and differentiation impairment of Tg2576 adult neural stem cells correlates with high levels of APP and AβOs. **a** Phase-contrast micrograph of aNSCs culture showing less primary neurospheres in Tg2576, that are also smaller in size, compared with WT. Scale bar 50 µm, ×10 magnification. Quantification of the number of primary neurospheres and of their size from single SVZ are expressed as mean  ± SEM of five animals (*n* = 5) for each experimental group. *P < 0.05, significantly different from WT, Mann–Whitney test. **b** Quantification of Sox2 and DCX positive cells in SVZ-derived neurospheres from Tg2576 (red) and WT (blue) animals. In vitro analyses show a reduction of aNSCs compartment (Sox2^+^ cells) and a significant increase in DCX^+^ neuroblasts, confirming the in vivo data. Data are means  ±SEM of five individual animals (*n* = 5) for each genotype. *P *<* 0.05, ***P < 0.001, significantly different from WT, Mann–Whitney test. **c** Cell proliferation is reduced in Tg2576 progenitors. Growth curves at early passages shows a significant decrease in proliferation of Tg2576 progenitors (red line) compared with WT sample (blue line). Quantification are expressed as mean  ± SEM of two pools of five animals for each experimental group examined in three independent experiments (*n* = 6). **P < 0.01, significantly different from WT, Mann–Whitney test. **d** Aβ and APP are highly expressed in Tg2576 SVZ and SVZ-derived neurospheres, as revealed by immunofluorescence staining for Aβ42 in SVZ (anti-Aβ 12F4) and human Aβ/APP (anti-hAβ/hAPP D54D2). Double immunostaining for hAPP/hAβ (green signal) and progenitors or neuroblasts markers (red signal) shows hAPP/hAβ generation in both Sox2^+^ progenitors and DCX^+^ neuroblasts in Tg2576 but not in WT samples. Scale bar 50 µm, ×40 magnification, zoom 1.9 (for SVZ) and 10 µm, ×63 magnification, zoom 2.5 (for neurospheres). White squared boxes in top panels represent ×2 magnification of the corresponding dot-lines insets. DAPI staining on nuclei in blue. Quantification of immunofluorescence intensity of APP/Aβ-expressing cells (*n* = 50) in neurospheres (NS), progenitors, and neuroblasts, as mean ± SEM of the analysis of three independent experiments. **P < 0.01, significantly different from WT, Mann–Whitney test. **e** Western blot (anti-Aβ WO2) and dot blot (DB) analysis of Tg2576 (Tg1 and Tg2) and WT (WT1 and WT2) total lysates demonstrates that human APP, APP C-99, and AβOs are almost exclusively detected in Tg2576 samples, as quantified in the histograms on the right. Comparable results are obtained by western blot analysis of conditioned media (**f**), in which s-APP and Aβ monomers (4 KDa) are detectable only in Tg2576 media, as quantified in the as quantified in the histogram on the right. Quantifications are reported in comparison with WT samples (at 1), obtained from densitometric values of bands and spots normalized for β-actin (cell lysates), for one band of ponceau (conditioned media), or for the corresponding ponceau staining of the dot (DB). Data are mean  ± SEM of two samples for each experimental group examined in three independent experiments (*n* = 6). *P < 0.05, ***P < 0.001 significantly different from WT, Student’s *t*-test

The neurogenic defects of OB interneurons in Tg2576 could reflect impairment in progenitor proliferation and/or in the neuronal maturation of SVZ neuroblasts. To test these hypotheses, we evaluated the proliferative and differentiative properties of neural progenitors derived from the adult SVZ region of 1.5-month-old Tg2576 and WT mice, grown as neurosphere cultures. The overall number of primary neurospheres obtained from Tg2576 mice was significantly lower than that from WT animals (Fig. 2a) and their size was smaller (average diameter of neurospheres: Tg2576 33.72 ± 3.9 μm; WT 58 ± 7.7 μm, Fig. 2a). This could reflect either a proliferative impairment of the SVZ progenitors and/or a reduction in the number of aNSCs resident in the SVZ, as observed in vivo. Indeed, Tg2576 neurospheres contained more DCX^+^ neuroblasts and less Sox2 progenitors (Fig. 2b), in agreement with the in vivo data. To explore also the second hypothesis we performed proliferation analysis on two Tg2576 and two WT neurospheres cultures (namely Tg1, Tg2 and WT1, WT2), obtained by pooling the primary neurospheres from five SVZ dissections for each genotype. Growth curves at early passages showed that Tg2576 aNSCs proliferated significantly less than WT progenitors (Fig. 2c). These results have been confirmed by double immunostaining for the pan-proliferative marker Ki67 and BrdU 24 h after in vitro BrdU labeling. The percentage of proliferating Ki67^+^ and Ki67^+^ BrdU^+^ double positive cells was significantly lower in Tg2576 respect to WT (Supplementary Fig. S1B), suggesting a slower cell cycle rate of Tg2576 cells, also because no significant difference was found between the two populations in terms of number of apoptotic cells, measured by activated-caspase-3 expression (Supplementary Fig. S1C).

Despite the absence of amyloid-plaques deposition in the Tg2576 brain at this early age [27], we investigated Aβ production in resident and SVZ-derived aNSCs. Immunostaining of brain sections of 1.5-month-old Tg2576 mice with 12F4 antibody (recognizing the C-terminal specific Aβ_42_ neo-epitope) shows that Aβ_42_ is abundant along the entire SVZ (Fig. 2d). As well, immunostaining on primary neurospheres with D54D2 antibody (preferentially recognizing human APP (hAPP) and human Aβ (hAβ) with respect to the endogenous mouse counterpart) confirmed that Tg2576 neurospheres express the transgenic hAPP and produce hAβ also in vitro (Fig. 2d). hAPP/Aβ are highly detected in both Sox2^+^ progenitors and DCX^+^ neuroblasts (Fig. 2d). Western blot analysis shows high levels of full length APP and APP-C99 terminal fragment in Tg2576 neurospheres cell extracts (Fig. 2e), and high levels of soluble-APP and Aβ monomers in Tg2576 neurospheres-derived conditioned media (Fig. 2f). Thus, the amyloidogenic pathway is maintained in Tg2576 neurospheres cells. Dot-blot (DB) analysis in native conditions demonstrates that Tg2576 neurospheres produce high levels of intracellular Aβ_42_, compared with WT cells. Notably, DB with the conformational Aβ oligomeric-specific scFvA13 antibody [24] shows an overproduction of intracellular AβOs in Tg2576 SVZ neurospheres (Fig. 2e). Instead, other generic amyloid conformers (such as generic prefibrillar and fibrillar oligomers, recognized by A11 and OC antibodies, respectively) did not change (Supplementary Fig. S2).

**Fig. 3:**
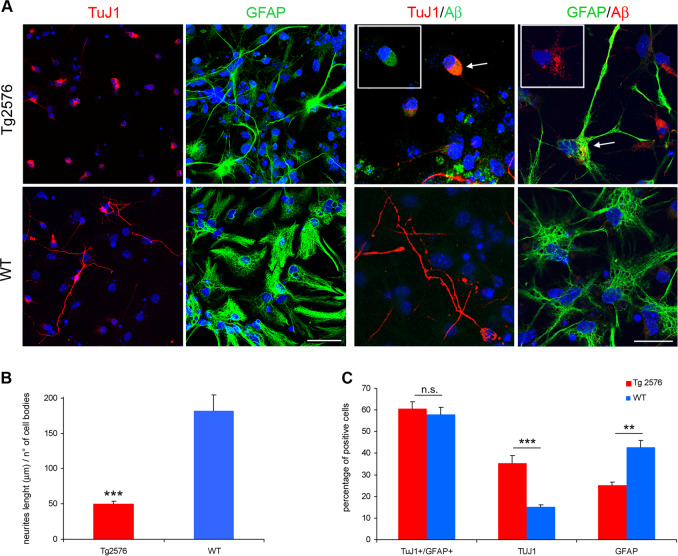
Differentiation impairment of Tg2576 aNSCs. **a** Immunostaining for TuJ1 (red) and GFAP (green) shows that Tg2576 progenitors fail to differentiate properly, as shown by the drastic reduction of neurites and the dystrophic cell shape of astrocytes. Both Tg2576 neurons and astrocytes express higher level of Aβ than their control counterpart (right panels, green and red signal, respectively, in the white squared boxes with the corresponding cell indicated by the arrows). Scale bar 50 μm, ×40 magnification, zoom 1.5 for the left panels and scale bar 20 μm, ×63 magnification, zoom 2 for the other panels. **b** Quantification of neurites length per number of neuron bodies in Tg2576 and WT  samples is expressed as mean  ± SEM of two samples for each experimental group examined in three independent experiments (*n* = 6). ***P < 0.001 significantly different from WT, Anova test. **c** Tg2576 differentiating neurospheres give rise to more TuJ1^+^ neurons at the expense of GFAP^+^ astrocytes. Quantification of percentage of positive TuJ1^+^ or GFAP^+^ cells in Tg2576 and WT samples is expressed as mean  ± SEM of two samples for each exerimental groups examined in three independent experiments (*n* = 6). **P < 0.01 (for GFAP^+^ astrocytes), ***P < 0.001 (for TuJ1^+^ neurons) significantly different from WT, Mann–Whitney test. n.s., not significantly different from WT, Mann-Whitney test

In addition to the proliferative defect of Tg2576 SVZ progenitors, we investigated a possible impairment of their differentiation in vitro into olfactory interneurons. To this aim, we measured by immunofluorescence the expression of the neuron-specific class III beta-tubulin (TuJ1) and of the astrocytes GFAP markers, upon neurosphere dissociation and differentiation. We showed that Tg2576 neurons are poorly differentiated (Fig. 3a, b), with very short neuritic processes. GFAP^+^ cells displayed an altered cell shape, as they lose the bushy-like morphology, typical of astrocytes, and displayed an elongated shape with few ramifications (Fig. 3a). This morphological phenotype was also observed in vivo in the SVZ of Tg2576 mice (Supplementary Fig. S3). Moreover, the majority of the Tg2576 progenitors produced TuJ1^+^ cells, while WT cells gave rise mainly to GFAP+ astrocytes (Fig. 3c). The increased number of TuJ1^+^ cells in Tg2576 cultures well correlates with the higher number of DCX^+^ neuroblasts found in vivo, and occurs at the expense of the glial compartment, as the number of GFAP^+^ cells is reduced in Tg2576, whereas the percentage of the overall differentiated cells (neurons and astrocytes) does not change between transgenic and WT animals (60.8 vs. 57.5%, Fig. 3c). This strongly suggests that both neuronal and glial defects observed in the differentiated neurospheres cultures are due to an intrinsic defect of the SVZ progenitors, probably caused by Aβ overproduction.

To test this hypothesis, we first analyzed the amount of Aβ in aNSCs-derived neurons and astrocytes, by double immunostaining for TuJ1 or GFAP and Aβ. Both Tg2576 neurons and astrocytes showed high level of Aβ, while WT differentiated cells were not immunoreactive for the D54D2 anti-hAβ antibody (Fig. 3a, right panels). We conclude that Aβ/AβΟs accumulation in progenitors and differentiated Tg2576 cells correlates with their proliferation and differentiation defects, suggesting a cell-autonomous defect.

### Conformational selective interference with intracellular Aβ oligomers rescues proliferative and differentiation defects of Tg2576 aNSCs

**Fig. 4:**
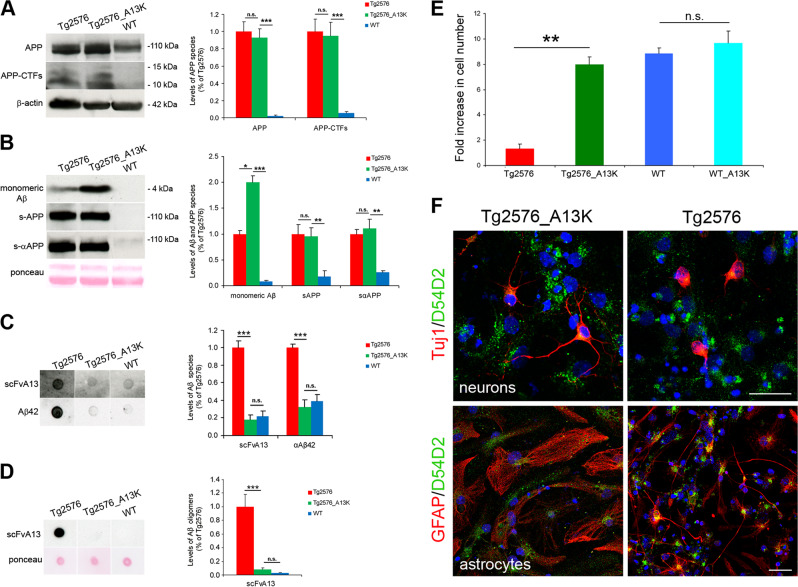
scFvA13-KDEL intrabody rescues the proliferative and differentiation impairment of Tg2576 aNSCs. **a**, **b** Western blot (WB) for full-length APP (anti-APP C-terminal) and for APP-CTFs, as well as for soluble APP (s-APP, anti-APP WO2) and soluble α-APP (sα-APP, anti-APP N-terminal 22C11) in Tg2576, WT and Tg2576_A13K lysates (**a**) and conditioned media (**b**) indicates that scFvA13 does not affect the processing of APP in the aNSCs, as quantified by the densitometric values reported in the corresponding histograms on the right. Dot Blot (DB) analysis on lysates (**c**) and conditioned media (**d**) shows that the stable expression of scFvA13-KDEL intrabody reduces the amount of AβOs to levels comparable to those measured in WT samples. The histograms shows the levels of Aβ species in WT, Tg2576 and Tg2576_A13K lysates and CM in comparison with Tg2576 samples (at 1), obtained from densitometric values of bands and normalized for β-actin (for lysates, **a** and **c**) or ponceau (for conditioned media, **b** and **d**). Data are mean  ± SEM of two samples for each experimental group examined in three independent experiments (*n* = 6). *P < 0.05, **P < 0.01, ***P < 0.001 significantly different from WT, Student’s *t*-test. n.s. not significantly different from Tg2576 or WT, Student's *t*-test. **e** Fold increase in cell number of aNSCs (Tg2576, Tg2576_A13K, WT, and WT_A13K) shows that the stable expression of scFvA13-KDEL intrabody leads to an undeniable rescue of the proliferative defect of Tg2576 progenitors, while does not influence proliferation of WT cells. Difference in proliferation rates has expressed as fold increase in cell number (FI), as mean  ± SEM of two samples for each experimental group examined in three independent experiments (*n* = 6). **P < 0.01, significantly different from Tg2576, Mann–Whitney test. n.s., not significantly different from WT, Mann–Whitney test. **f** Immunofluorescence staining with D54D2 antibody for hAβ42/hAPP (green) and TuJ1 or GFAP (red) shows that in Tg2576_A13K differentiated cells there is a partial rescue of the dystrophic shape of astrocytes, while a more robust rescue of neurons maturation and arborization is evident, despite the Aβ production. Scale bar 25 μm, ×63 magnification, zoom 1.3 and 2.5 (neurons and astrocytes panels, respectively)

We have previously demonstrated that the intracellular expression of the scFvA13 anti-AβOs intrabody in the ER (scFvA13-KDEL) allows a CSI with AβOs pools but not with Aβ monomers nor with APP processing or trafficking [24]. The scFvA13-KDEL intrabody targets intracellularly formed AβOs in the ER, interfering with their endogenous formation and actions [24]. In order to investigate if endogenous human AβOs were responsible for the proliferative and differentiative impairment of Tg2576 progenitors in a cell-autonomous manner, we expressed in these cells the scFvA13-KDEL intrabody [24] via lentiviral infection (pLentiA13K_GFP lentivirus, see Supplementary Materials and methods, and Supplementary Fig. S4A). The intrabody was detected in infected (GFP positive) progenitors, differentiated neurons, and astrocytes by immunofluorescence staining for its C-terminal V5 tag (Supplementary Fig. S4B). The subcellular localization of scFvA13-KDEL in the ER was demonstrated by double immunostaining for V5 and the ER marker Calnexin (red and green signal, respectively, in Supplementary Fig. S4B), and its enrichment in the ER was confirmed by immunoprecipitation analysis of microsome extracts (Supplementary Fig. S4C). WB analysis showed that the steady-state levels of full-length APP and of APP C-terminal fragments (CTFs) in total lysates (Fig. 4a), as well as of soluble α-APP and β-APP in the extracellular medium (Fig. 4b), are similar in Tg2576 and Tg2576 infected (Tg2576_A13K) progenitors. Moreover, DB analysis showed that in Tg2576_A13K cells the intrabody expression significantly and selectively reduced the levels of intracellular Aβ42 and scFvA13-positive AβOs (Fig. 4c) and of extracellular scFvA13-positive AβOs (Fig. 4d) to those measured in WT progenitors. Other generic amyloid conformers (such as generic prefibrillar oligomers and fibrillar oligomers, recognized by A11 and OC antibodies, respectively) remained unchanged (Supplementary Fig. S4D). Of note, these results in the Tg2576 neurospheres model provide a further example of CSI against AβOs, mediated by scFvA13-KDEL intrabody [24].

Strikingly, scFvA13-KDEL expression rescued both the proliferative and the differentiation defects of Tg2576 progenitors. Cell proliferation of Tg2576_A13K, analyzed by growth curves, Ki67 expression and BrdU incorporation, was comparable to that of WT cells (Fig. 4e and Supplementary Fig. S5A), and the rescue by the intrabody did not depend on a difference in the percentage of apoptotic cells (Supplementary Fig. S5B). Both glial and neuronal morphology of Tg2576_A13K differentiated progenitors (GFP^+^, Supplementary Fig. S5C) was restored to the WT phenotype, despite the persistent expression of Aβ in the culture (Fig. 4f). The effect of scFvA13-KDEL intrabody was particularly appreciable in neuronal differentiation, since neurites length of Tg2576_A13K neurons was identical to that of WT cells (Supplementary Fig. S6).

### Morphological differentiation defects of Tg2576 neurons are due to AβO-dependent hyperstabilization of microtubules

**Fig. 5:**
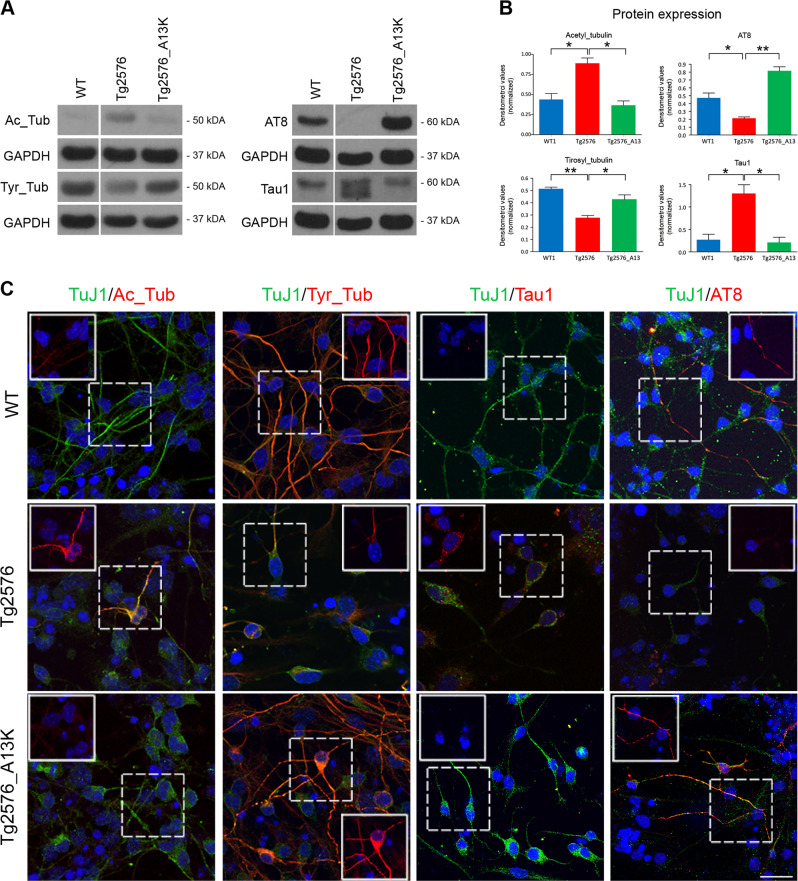
Rescue of cytoskeleton alterations in Tg2576_A13K aNSCs. **a** Western blot analysis of Tg2576, Tg2576_A13K and WT cell lysates with antibodies against the Tau-1/AT8 epitopes and against acetyl (stable)- and tyrosinylated (instable)- α−tubulin. Tg2576 aNSCs display hyperstable microtubules, as indicated by the dramatic downregulation in AT8 Tau immunoreactivity (left panel) and the important upregulation in acetyl tubulin-positive microtubules (right panel). Interestingly, stable expression of scFvA13 intrabody is able to significantly rescue the cytoskeleton alteration, normalizing the microtubule instability to physiological control levels. **b** Densitometric quantification of the AT8, Tau1, acetyl-α−tubulin, and tyrosinylated-α−tubulin intensity bands in WT (blue), Tg2576 (red), and Tg2576_A13K (green) samples was calculated normalizing for GAPDH used as loading internal control. Data are means  ± SEM of two samples for each experimental group examined in three independent experiments (*n* = 6). *P < 0.05, **P < 0.01, significantly different from WT, Student’s *t-*test. **c** Immunofluorescence staining for neurons (TuJ1, green signal) and the two forms of α−tubulin or Tau-1/AT8 epitopes (red signal) confirms the WB results. The higher level of acetyl-α−tubulin and of phosphorylated tau in Tg2576 samples are rescued at level comparable to those measured in WT by the stable expression of anti-AβOs intrabody. DAPI staining on nuclei in blue. Scale bar 25 μm, ×63 magnification, zoom 1.5. White squared boxes represent only the red signal of the corresponding dot-lines insets

Neurons derived from Tg2576 neurospheres are poorly differentiated, with very short neurites (Fig. 3a). Neuronal morphology relies on the organization of the cytoskeleton and the microtubule-associated-protein(s) tau regulates microtubule dynamics in neurons [29, 30]. We investigated the dynamic state of microtubule network in WT, Tg2576, and Tg2576_A13K neurospheres by evaluating the site-specific phosphorylation of tau, and the related acetylation and tyrosinylation of α-tubulin, as markers of microtubule stability [31]. WB analysis shows a dramatic downregulation of phospho-tau (AT8), with an inverse increase in reciprocal non-phospho Tau-1 immunoreactivity in Tg2576 aNSCs, compared with WT (Fig. 5a, b). As well, Tg2576 aNSCs display a significant upregulation of acetyl-αtubulin (stable) with an inverse downregulation of tyrosinylated-α−tubulin (instable). The overall results demonstrate a pattern of hyperstabilized microtubules. Interestingly, scFvA13-KDEL antibody expression in Tg2576 neurospheres induces a massive increase of tau phosphorylation (AT8) coupled with a significant decrease of Tau-1. Moreover, we observed a significant decrease of acetylation of tubulin mirrored by an increase of its tyrosinylation. Thus, the selective intrabody interference with hAβO normalized the microtubule instability to physiological control levels, by modulating the endogenous murine tau and tubulin. Immunofluorescence staining for the two forms of α−tubulin and the Tau-1/AT8 tau epitopes confirmed the WB results (Fig. 5c). As a consequence of the microtubule instability recovery, Tg2576_A13K neurospheres gave rise to fully differentiated Tuj1^+^ positive neurons, whose neurites length was comparable to that of control neurons (Fig. 5c). In conclusion, we report the first evidence that morphological defects of Tg2576 neurons, depending on cytoskeleton alterations already present in aNSCs, are driven upstream by human endogenous AβOs, in a tau- and tubulin-dependent manner, since they are rescued by AβO-specific CSI.

### In vivo delivery of scFvA13-KDEL intrabody rescues aNSCs proliferation and olfactory bulb neurogenesis

**Fig. 6:**
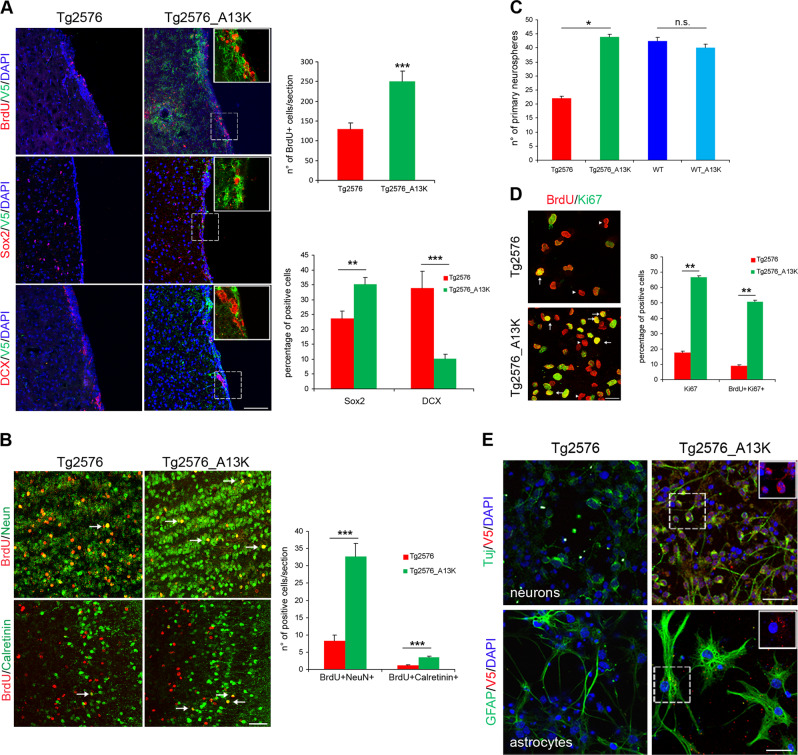
In vivo rescue of Tg2576 impaired neurogenesis by scFvA13-KDEL lentiviral infection. **a** Lentiviral delivery of scFvA13-KDEL intrabody to Tg2576 SVZ. The intrabody expression, confirmed by immunofluorescence staining for its C-terminal tag V5 (in green, right panels), leads to a significant rescue of proliferation, demonstrated by the increased number of BrdU^+^ cells (red signal), and restores the correct number of progenitors (Sox2^+^, red signal in central right panel) and neuroblasts (DCX^+^, red signal in bottom right panel) in the infected SVZ. DAPI staining on nuclei in blue. Scale bar 75 µm, ×40 magnification. White squared boxes in top panels represent ×2.5 magnification of the corresponding dot-lines insets. The histograms represent the quantification of BrdU or of Sox2 and DCX positive cells in Tg2576 (red) and Tg2576_A13K (green) SVZ. **b** Immunostaining for BrdU and NeuN (red and green, respectively, top panels) and BrdU and Calretinin (red and green, respectively, bottom panels) in the OB of infected animals shows a significant increase of newborn Calretinin interneurons derived from Tg2576_A13K SVZ progenitors compared with Tg2576, as quantified in the histogram. Scale bar 50 µm, ×40 magnification. **c** Quantification of the number of primary neurospheres derived from Tg2576 (red) and Tg2576_A13K (green) SVZ show ex-vivo rescue of proliferation by the in vivo intrabody expression. **d** Immunofluorescence staining for BrdU and Ki67 (red and green signals, respectively, in left panels), showing more BrdU^+^ (arrowheads) and double BrdU^+^Ki67^+^ (arrows) cells in Tg2576_A13K samples compared wth their noninfected counterpart. DAPI staining on nuclei in blue. Scale bar 25 µm, ×63 magnification. **e** Immunofluorescence staining for neurons (TuJ1, green signal in upper panels) and astrocytes (GFAP, green signal in lower panels) shows that the expression of scFvA13 intrabody (V5^+^ cells, red signal) leads to a partial rescue of both the neuronal arborization and the dystrophic cellular shape of astrocytes. DAPI staining on nuclei in blue. Scale bar 25 μm, ×63 magnification, zoom 1.5. White squared boxes represent only the V5 signal (in red) of the corresponding cells dot-lines insets. Data are means ± SEM of five individual animals (*n* = 5) for each experimental group. *P < 0.05, **P < 0.01, ***P < 0.001, significantly different from Tg2576, Mann-Whitney test. n.s., not significantly different from WT, Mann-Whitney test

To test the hypothesis whether endogenous AβOs are responsible for the proliferative and differentiation impairment of Tg2576 progenitors also in vivo, we performed intracerebral delivery of pLentiA13K-GFP virus into the SVZ of 1.5-month-old Tg2576 mice, through stereotaxic injection. The intrabody expression in Tg2576 progenitors in the SVZ in vivo rescues their proliferation, measured by number of BrdU^+^ and Sox2^+^ cells, while the number of DCX^+^ neuroblasts was lower compared with non-infected Tg2576 mice (Fig. 6a). This latter result well correlates with an increase in the olfactory bulb neurogenesis, as demonstrated by a higher number of BrdU^+^NeuN^+^ and BrdU^+^Calretinin^+^ newborn neurons in the OB (Fig. 6b). Moreover, intrabody expression in Tg2576 SVZ increases the number of primary neurospheres to that obtained from WT SVZ (Fig. 6c). Ex-vivo analysis of primary neurospheres, derived from infected and controlateral non-infected SVZ, confirmed that the expression of scFvA13-KDEL persists also in vitro (Supplementary Fig. S7A) and led to the same rescue, previously demonstrated in vitro by lentiviral infection, of both proliferation and differentiation defects of the SVZ progenitors (Fig. 6d, e). Moreover, the cell type composition of SVZ_A13K-derived neurospheres reflected the in vivo data (Supplementary Fig. S7B). Thus, the intracellular interference with AβOs in vivo, through the delivery of scFvA13-KDEL intrabody, restores a correct SVZ AN.

In conclusion, our data demonstrated that impaired AN is an early event occurring in the SVZ neurogenic niche of young Tg2576 mice, prior to Aβ plaques deposition and overt neurodegeneration, but dependent on the intracellular generation and accumulation of toxic endogenous AβOs in SVZ aNSCs. We also demonstrated the effectiveness in vivo of an innovative disease-modifying approach, based on an intrabody gene therapy strategy selective for AβOs.

## Discussion

In this work, we demonstrated a severe impairment of SVZ AN in young Tg2576 mice, the earliest event observed in the neurodegeneration progression in this AD model [26], and formally proved that this deficit is triggered by intracellular AβOs. Specifically, we provided formal evidence of a causal link between the intracellular formation of toxic natural-occurring hAβOs in aNSCs and altered neurogenesis occurring prior to neurodegeneration. An independent study showed defects in DG neurogenesis in 3-months-old Tg2576 [32], but no characterization of the Aβ/AβOs biochemical profiles was performed. Here we characterized the different intracellular/extracellular Aβ/AβO species present in Tg2576 aNSCs using combinations of different antibodies, especially conformational ones. We provided the first demonstration that intracellular AβOs generated within aNSCs are responsible for the proliferative impairment and the neurogenic defects of these cells, as well as for the morphological defects of neurons and astrocytes generated from these progenitors. The causal role of intracellularly formed AβOs in determining the neurogenesis defects has been formally demonstrated by the intracellular interception of AβOs by the scFvA13 intrabody in the ER of aNSCs, which reestablished proper neuronal and glial differentiation. This is a significant novel finding, as we exploited the approach of intracellular targeting and CSI with AβOs [24] in aNSCs. Moreover, previous studies either did not discriminate between the different Aβ species (due to the use of anti-Aβ antibodies that recognize several Aβ/APP isoforms) [18, 20], or used synthetic, rather than naturally occurring, and more bioactive Aβ_42_ peptides [21–23, 33]. Instead, scFvA13-KDEL intrabody specifically intercepts endogenous, naturally formed, biologically active AβOs conformers [24, 34] and lead to their functional silencing, in vitro and, notably, in vivo.

Neuronal differentiation of Tg2576 progenitors is greatly impaired, both in vitro and in vivo, despite the higher number of neuroblasts found in the SVZ. Exogenously applied Aβ_1–42_ drives NSCs of the SVZ toward a neuronal lineage [35]. Our results with endogenous Aβ confirmed this bias towards the neuronal lineage, which is however accompanied by a block in maturation of Tg2576 neurons.

In addition to the neurogenic defects, we also found a severe morphological alteration of GFAP+ astrocytes, which show an atypical elongated shape, both in the differentiating Tg2576 neurospheres in vitro and in vivo in the SVZ. This phenotype is reminiscent of the changes in astrocytes morphology observed in AD models [36, 37] and of the neurotoxic reactive A1 astrocytes phenotype [38]. We demonstrate that the morphological alteration of astrocytes is causally linked to the presence of intracellular (and possibly secreted) AβOs in the aNSCs.

The morphological defects observed in Tg2576 neurons and astrocytes are likely due to tau-mediated cytoskeleton abnormalities, which are already present in the neural stem cells compartment. We find that Tg2576 neurospheres display hyperstabilized microtubules, as indicated by tau dephosphorylation and the important upregulation in acetyl tubulin-positive microtubules. Significantly, the conformational-dependent targeting of intracellularly formed AβOs reverts these events. As consequence, a correct cell morphology of neurons and astrocytes is restored. We thus provide a new mechanism of microtubule destabilization driven upstream by human AβOs, through the post-translational modulation of tau and tubulin, extending previous findings in AD neuronal models [39, 40].

The role of SVZ neurogenesis in AD pathogenesis has been less investigated [8], compared with hippocampal neurogenesis. Our results prospect an impaired SVZ neurogenesis as an evolutive biomarker of AD neurodegeneration in humans. This strongly supports further analysis of SVZ neurogenesis both in AD animal models and human patients, where in vivo imaging reagents to noninvasively label SVZ neurogenesis might be proposed. Our finding prompts to investigate systematically whether AN markers in the neurogenic niche(s) represent an early biomarker also in human AD brains. Markers for AN in the human CSF [41] could serve as proxies for the rates of neurogenesis in the brain. A CSF biomarker reflecting the extent of early alterations of neurogenesis in MCI or in nondemented patients with memory loss would be extremely valuable for longitudinal studies, for early diagnosis and patient stratification, or for monitoring treatment efficacy.

Moreover, strategies aimed at restoring or reinforcing AN might represent complementary therapeutic interventions for AD. Of note, increased neurogenesis has been reported in nondemented individuals with AD neuropathology compared to demented AD cases [42], suggesting that neurogenesis might protect against disease progression. Our demonstration that the action of AβOs in inducing alterations of SVZ neurogenesis is cell-autonomous provides new evidence to the concept of intracellularly formed AβOs as a key target for AD therapy [24, 43, 44]. Thus, targeting intracellular AβOs in SVZ can be prospected as a future therapeutic strategy. Of note, in humans SVZ neurogenesis contributes substantially to the interneurons population in the striatum [2], which is commonly associated with motor function, procedural learning, and memory, as well as with cognitive flexibility [45]. Interestingly, loss of dopaminergic neurons in the ventral tegmental area of the striatum has been proposed to contribute to AD pathogenesis at early stages [46]. In this view, strategies aiming at reinforcing SVZ-dependent striatal neurogenesis in human might contribute to counteract the disease progression.

Our finding has strong translational implications, as it identifies impaired SVZ neurogenesis as a novel biomarker for early AD diagnosis. Remarkably, a very recent paper reported that neurogenesis is impaired in AD patients already at the earliest Braak stage, before amyloid-plaque formation [7], providing a strong cross-validation with our animal model study. On the other hand, our finding provides a mechanism, formally proving that intracellular AβOs in the ER of SVZ progenitors is responsible for the observed defects, an outcome that can be prospectively extended to the human AD case. Future investigations are required in humans, possibly combining available Aβ imaging tools (i.e., PIB-PET) and MRI-based assessment of neurogenesis [47, 48].

In addition, we demonstrate for the first time that targeting intracellular AβOs in aNSCs, by a gene-therapy approach, might provide a new selective therapeutic strategy to neutralize intracellular natural-occurring AβOs and, at the same time, to restore a functional neurogenesis.

## Supplementary information

Supplemental Information
