# Supplementary material for: Impaired adult neurogenesis is an early event in Alzheimer’s disease neurodegeneration, mediated by intracellular Aβ oligomers
Source: Cell Death Differ. 2019 Oct 7;27(3):934–48. doi: 10.1038/s41418-019-0409-3 (PMC7206128; doi:10.1038/s41418-019-0409-3)
Supplement: Supplementary file 1 — Supplemental Information [file 41418_2019_409_MOESM1_ESM.pdf]

Supplemental Information

Supplementary Materials and methods

Matrigel coating

Matrigel (BD Biosciences, NY, USA) was diluted 1:100 in sterile water at 4°C. This solution was used to coat 12 mm diameter coverslips by incubating for 2 h at 37°C. After incubation, matrigel was removed and coverslips were washed three times with sterile water before use.

Cell Number Quantification

Stereological analysis of the number of cells in the SVZ and in the olfactory bulbs were obtained by counting cells visualized with confocal microscopy throughout the whole rostro-caudal extent of these structures in one-in-six series of 40-µm free-floating coronal sections. To obtain the average number of SVZ or OB cells per area, the number of positive cells for each SVZ and OB section was then divided by the total number of sections counted (5-6). Five animals per group (n = 5) were analyzed. Statistical analysis was performed by Mann-Whitney test, and all experiments were expressed as mean ± SEM.

To quantify the number of differentiated cells, the number of Tuj1 and glial fibrillary acid protein (GFAP) immunoreactive cells was counted in at least 10 non-overlapping fields in each sample, for a total of >1000 cells per sample. The total number of cells in each field was determined by counterstaining cell nuclei with DAPI (Sigma-Aldrich, D9542,

50 mg/ml in PBS for 15 min at RT). The average percentage of differentiated cells for each sample was then calculated by dividing the number of Tuj1 and GFAP positive cells by the total number of cells for each field. Data are the mean ± SEM of two independent samples for each experimental group examined in three independent experiments (n = 6). To quantify neuritic processes, the length of Tuj1-labeled filaments was measured in 7 non-overlapping fields through semi-automatic filament count using IMARIS software. Filament seed points were manually added by an experimenter blind to group samples. Total filament length in each field was divided by the number of DAPI-positive cells and expressed as average density of neurites ramification per cell.

Sample preparation for biochemical analysis

For conditioned medium (CM) preparation, cells were grown up to ~90% confluence, then CM was collected and cleared of cell debris by centrifugation at 200g for 10 min. Ponceau S staining of nitrocellulose membrane before western blot (WB) and dot blot (DB), which detect the total proteins, was used as normalization of WB lanes loading and DB spots.

Cell lysates were prepared in Tris-HCl 50 mM pH 8, NaCl 150mM, NP40 1% with the addition of cocktail of proteases and phosphatases inhibitors (Roche). The lysates were centrifuged at 13000 rpm for 20 min. The supernatant was collected and the concentration of total proteins was determined by BCA assay. In this case, the β-actin staining was used as normalization of WB lanes loading.

Sample preparation and Western blot analysis for cytoskeleton

Cell lysates were prepared as described before. The supernatant was then collected and the amount of total protein was determined by Bradford assay (Protein Assay Dye Reagent Concentrate, BioRad). Equal amounts of protein were separated by 10 and 12% SDS–PAGE and transferred to nitrocellulose membrane filters 0.45μm (GE Healthcare, 10600002). The filters were blocked in TBS-T containing 5% non-fat dried milk for 1h at room temperature or overnight at 4°C. Proteins were visualized using appropriate primary antibodies. All primary antibodies were diluted in TBS-T and incubated with the nitrocellulose blot overnight at 4°C. After incubation with secondary peroxidase coupled anti-mouse, anti-rabbit or anti-goat antibodies, chemiluminescent detection was performed by using the ECL system (SuperSignal West Pico, ThermoFisher Scientific, 34080; ECL Prime, GE Healthcare, RPN2232). Protein loading was monitored by normalization to GAPDH level. Blots were scanned and quantitative densitometric analysis was performed by using ImageJ software (http://imagej.nih.gov/ij/) as described below.

Quantitative densitometric analysis

Films were scanned at high resolution (600 dpi) with a Nikon scanner in TIFF file format. Acquired images (in "Greyscale" mode) were analyzed by ImageJ software. For multiple proteins analysis, the same region of interest (ROI) was defined for each row of protein bands or dots across the lanes. The ROI size covered the minimum area to contain the whole of the largest band or dot of the row. Selected bands/dots and their backgrounds (framed with the same ROI of bands/dots) were then measured by ImageJ software using the "measure" function. Loading controls (actin or ponceau) measurements were taken in

the same way for the bands/dots. Data were exported into a Microsoft Excel and net bands/dots and loading controls were calculated by deducting the background from the band/dot value. The final relative quantification values are the ratio of net band to net loading control.

Microsome extraction

Cells were pelleted and resuspended in homogenization buffer (10 mM HEPES, pH 7.4, 1 mM EDTA and 0.25 M sucrose) supplemented with protease inhibitor cocktail (Sigma-Aldrich). Cells were disrupted with a Dounce homogenizer (10 strokes) followed by five passages through a 27-gauge needle. After that, homogenized cells were centrifuged for 10 min at 1500 g. Nuclei and unbroken cells were pelleted and then resuspended in homogenization buffer. The cells were disrupted as described before and then were centrifuged for 10 min at 1500 g. The supernatants from both spin were pooled and then were centrifuged for 1 hour at 65000 g. The resultant vesicle pellets were the total microsomes.

Intrabody-AβOs co-IP from microsome preparations

Freshly prepared microsome preparations were broken by two step of freeze and thaw at -80°C. Broken microsomes were incubated with a cross-linker solution of dithiobis (succinimidyl propionate) (DSP, Pierce), to a final concentration of 2 mM for 30 min at RT, after that Stop Solution (1M Tris pH 7.5) was added to a final concentration of 20 mM for 15 minutes. Cross-linked microsomes were incubated with anti-V5 agarose affinity gel (Sigma-Aldrich) for 16 hours at 4°C for the immunoprecipitation procedure. After ice-cold PBS washes (6 times 5000 g, 5 min each), the immunoprecipitated proteins

were eluted with NuPage™ LDS sample buffer (ThermoFisher Scientific, NP0007) containing β-mercaptoethanol by 10 min boiling. Co-IP was analyzed in pre-casted NuPAGE™ Novex™ 10% Bis-Tris Midi Protein Gels (ThermoFisher Scientific, WG1201A), as described in Materials and Methods ("Western Blot Assay for Aβ" paragraph).

Lentiviral Vector Preparation and Neurospheres Infection

The scFvA13-KDEL cDNA was amplified from scFvA13Express vector (Meli *et al.*, 2014) by using adaptor primers to insert XbaI restriction site and cloned by restriction with XbaI enzyme into the pRRL-CMV-PGK-GFP-WPRE (TWEEN) vector. This bicistronic vector allows the simultaneous expression of the GFP and scFvA13KDEL. The lentivirus vector was prepared by the transient transfection of 293T cells using the calcium phosphate precipitation method. In particular, 293T cells were transfected with TWEEN vectors together with pMDL/pRRE, pMDG2.G and pRSV-Rev over night. The day after 293T cell medium was replaced and on day 1 and 2 of change medium supernatant, containing lentivirus vectors, was collected, pelleted, filtered and ultracentrifugated at 2600 rpm for 2 h at 4°C. The resulting pellet was re-suspended in 200 μl of HBSS, aliquoted and stored at -80°C for the following experiments. The titer of lentiviral vectors, measured by p24-test ELISA kit (Lenti-X™ p24 Rapid Titer Kit, Clontech, 632200), was 1.7×10^7 IFU/μl. 1 X 10^5 viable cells were plated in a T24 multiwell in growing medium and infected with 5 μl of TWEEN-A13KDEL lentiviral vector for 8 h. The day after neurospheres medium was replaced with fresh medium and 48 h after, viral infection was assessed by GFP expression at fluorescence microscope.

Stereotaxic surgery

Mice were anesthetized with 10 mg/kg solution of Zoletil (2.5 mg/ml) and Rompun (1 mg/ml) to obtain deep anesthesia and post surgery recovery. Upon assessment of deep anesthesia by pedal reflex, mice were mounted on stereotaxic apparatus located in a biosafety level 2 (BSL-2) room. Injecting cannulae were inserted into the sub-ventricular zone, SVZ (coordinates relative to bregma: +0.6 antero-posterior, +1 lateral and -2.7 ventral) injections were performed using a 10 µl hamilton syringe connected to a gauge injector. Mice were injected unilaterally with 1 µl of scFv_A13K lentiviral particles ($1.7X10^7$ IFU/µl), in one hemisphere while the opposite hemisphere was not injected and used as internal control. Hearth rate, respiratory rate and temperature were monitored during surgery procedure and until recovery of the animals. All surgical procedures were run between 9am and 1pm to allow monitoring of post-surgery recovery. Upon recovery, mice were kept in ventilated cages in groups of four littermates and sacrificed 10 days (for SVZ NSCs proliferation) or 31 days (for OB neurogenesis) later through perfusion in 4% PAF.

Table I. Antibodies Used

Pimary Antibodies	Specificity	Host species	Diluition	Supplier	Reference
BU1/75	BrdU	Rat monoclonal	ICH: 1:400	BioRad	MCA2060
SP6	Ki67	Rabbit monoclonal	ICH: 1:200	Abcam	ab16667
5A1E	cleaved caspase-3	Rabbit monoclonal	ICH: 1:1,000	Cell Signalling Technology	9664
12F4	Human, Mouse, Rat Aβ 1-42	Mouse monoclonal	ICH: 1:250	Biolegend	SIG-39142
Anti-NeuN	Neuronal nuclei (NueN)	Mouse monoclonal	ICH: 1:500	Merck Millipore	MAB377
Anti-glial fibrillary acid protein	Glial fibrillary acid protein (GFAP)	Rabbit polyclonal	ICH: 1:500	DakoCytomation	Z0334
C-19	glial fibrillary acid protein (GFAP)	Goat polyclonal	ICH: 1:300	Santa Cruz Biotechnology,	sc-6170
Y-17	Sox2	Goat polyclonal	ICH: 1:300	Santa Cruz Biotechnology	sc-17320
C-18	DCX	Goat polyclonal	ICH: 1:300	Santa Cruz Biotechnology	sc-8066
Anti-calretinin	Calretinin	Rabbit polyclonal	ICH: 1:300	Swant,	7699/4
C-20	Calbindin	Goat polyclonal	ICH: 1:1,000	Santa Cruz Biotechnology	sc-7691
TUJ1	Neuronal class III α-Tubulin	Mouse monoclonal	ICH: 1:250	Covance	MMS-435P
TUJ1	Neuronal class III α-Tubulin	Chicken polyclonal	ICH: 1:500	Abcam,	ab41489
D54D2	Human, mouse Aβ	Rabbit monoclonal	ICH: 1:200	Cell Signaling Technology,	8243
JL-8	Green Fluorescent Protein	Rabbit monoclonal	ICH: 1:500	Clontech	632381
6-11B-1	Acetylated Tubulin	Mouse monoclonal	ICH: 1:500 WB: 1:1500	Sigma-Aldrich	T7451

YL1/2	tubulin alpha	Rat monoclonal	ICH: 1:200 WB: 1:1000	BioRad	MCA77G
PC1C6	Tau1 Ser-195/Ser-198 epitopes	Mouse monoclonal	ICH: 1:200 WB: 1:1000	Merck Millipore	MAB3420
AT8	Phospho-PHF-tau pSer202+ Thr205	Mouse monoclonal	ICH: 1:200 WB: 1:500	ThermoFisher Scientific	MN1020
Anti-calnexin	calnexin	Rabbit polyclonal	ICH: 1:2,500	Sigma-Aldrich	C4731
Anti-V5 tag	V5 Tag	Mouse monoclonal	ICH and WB: 1:1,000	ThermoFischer Scientific	R960-25
WO2	Mouse and human Anti-Aβ	Mouse monoclonal	WB: 1:1,000	Merck Millipore	MABN10
Anti amyloid precursor protein	Rat, mouse and human APP C-terminal fragment	Rabbit polyclonal	WB: 1:3,000	Sigma-Aldrich	A8717
22C11	Mouse and human APP N-terminal	Mouse monoclonal	WB: 1:1,000	Merck Millipore	MAB348
AC74	α-Actin	Mouse monoclonal	WB: 1:5,000	Sigma-Aldrich	A2228
A11	Mouse, human and rat Aβ oligomers	Rabbit polyclonal	DB: 1:500	Merck Millipore	AB9234
scFvA13	Aβ oligomers				Meli *et al.*, 2014
OC	Human amyloid Fibrils	Rabbit polyclonal	DB: 1:1,000	Merck Millipore	AB2286
HIS.H8	His tag	Mouse monoclonal	DB: 1:2,000	Merck Millipore	05949
295F2	Human, rat and mouse Abeta42	Mouse monoclonal	DB: 1:1,000	Synaptic Sistems,	218721
6C5	GAPDH	Mouse monoclonal	WB: 1:4000	Santa Cruz Biotechnology	sc-32233

Secondary Antibodies	Specificity	Host species	Diluition	Supplier	Reference
Donkey-anti rabbit igg (H+L) conjugated to Alexa488	Rabbit IgG	Donkey polyclonal	ICH: 1:500	ThermoFisher Scientific	R37118
Donkey-anti rabbit igg (H+L) conjugated to Alexa594	Rabbit IgG	Donkey polyclonal	ICH: 1:500	ThermoFisher Scientific	R37119
Donkey-anti rabbit igg (H+L) Highly Cross-Absorbed conjugated to Alexa 647	Rabbit IgG	Donkey polyclonal	ICH: 1:500	ThermoFisher Scientific	A-31573
Donkey-anti mouse igg (H+L) Highly Cross-Absorbed conjugated to Alexa 488	Mouse IgG	Donkey polyclonal	ICH: 1:500	ThermoFisher Scientific	A-21202
Donkey-anti mouse igg (H+L) conjugated to Alexa 594	Mouse IgG	Donkey polyclonal	ICH: 1:500	ThermoFisher Scientific	R37115
Donkey-anti mouse igg (H+L) Highly Cross-Absorbed conjugated to Alexa 555	Mouse IgG	Donkey polyclonal	ICH: 1:500	ThermoFisher Scientific	A-31570
Donkey-anti mouse igg (H+L) Highly Cross-Absorbed conjugated to Alexa 647	Mouse IgG	Donkey polyclonal	ICH: 1:500	ThermoFisher Scientific	A-31571
Goat anti-rat conjugated to Alexa 594	Rat IgG	Donkey polyclonal	ICH: 1:500	ThermoFisher Scientific	A-11007
Donkey anti-goat antiserum conjugated to Alexa 488	Goat IgG	Donkey polyclonal	ICH: 1:500	ThermoFisher Scientific	A-11055

Donkey anti-goat antiserum conjugated to Alexa 594	Goat IgG	Donkey polyclonal	ICH: 1:500	ThermoFisher Scientific	A-11058
Donkey anti-goat antiserum conjugated to Alexa 555	Goat IgG	Donkey polyclonal	ICH: 1:500	ThermoFisher Scientific	A-21432
Donkey anti-goat antiserum conjugated to Alexa 647	Goat IgG	Donkey polyclonal	ICH: 1:500	ThermoFisher Scientific	A-21447
Donkey anti-chicken antiserum conjugated to Alexa 647	Chicken IgG	Donkey polyclonal	ICH: 1:250	ThermoFisher Scientific	A-21449
Anti-rabbit-HRP	IgG	Donkey polyclonal	WB and DB: 1:4000	Jackson ImmunoResearch	711-035-152
Anti-mouse-HRP	IgG	Donkey polyclonal	WB and DB: 1:4000	Jackson ImmunoResearch	715-035-151
Anti-mouse igG(whole molecule)-Peroxidase antibody	Mouse IgG	Goat polyclonal	WB: 1:10000	Sigma-Aldrich	A4416

Supplementary Figures and Legends

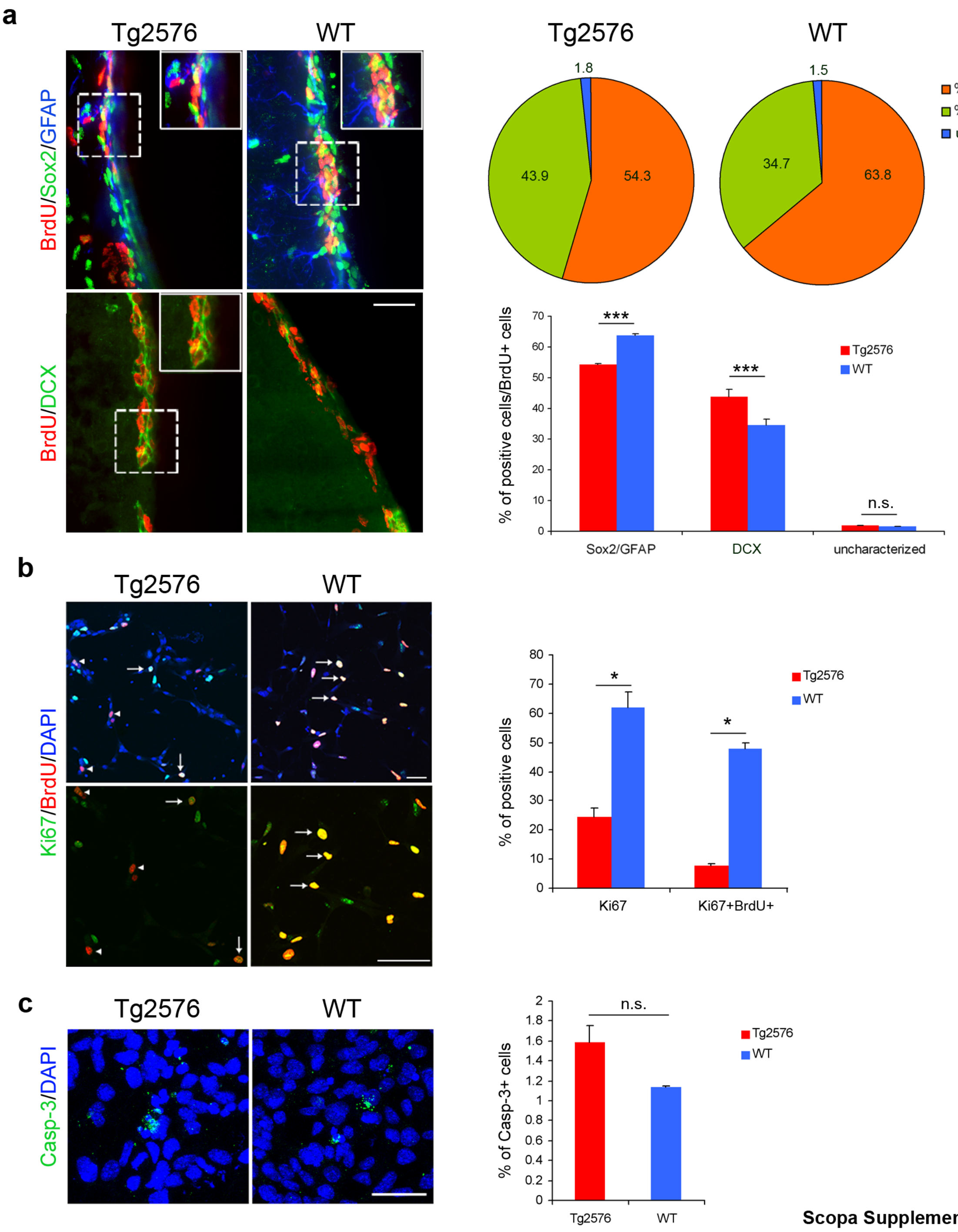

Scopa Supplementary Figure S1

Supplementary Figure S1. Proliferation and cell composition features of Tg2576 SVZ neurospheres.

(**A**) Characterization of BrdU incorporating cells in Tg2576 and WT SVZ by double immunofluorescence for BrdU (red signal) and progenitors (Sox2$^+$, green signal and GFAP$^+$, blue signal) or neuroblasts markers (DCX$^+$, green signal). While in WT SVZ the proliferating, BrdU$^+$ cells are mainly Sox2 and GFAP progenitors, in Tg2576 SVZ the BrdU$^+$ cells are equally distributed between progenitors and neuroblasts, as quantified in the pie charts. Scale bars 25 µm, 40X magnification. White squared boxes in top panels represent 2X magnification of the corresponding dot-lines insets. The histogram represents the quantification of BrdU$^+$/Sox2$^+$/GFAP$^+$ and BrdU$^+$/DCX$^+$ cells in Tg2576 (red) and control (blue) SVZ. Data are means ± SEM of five individual animals (n =5) for each experimental group. ***P < 0.001, significantly different from WT, Mann-Whitney test. n.s., not significantly different from WT, Mann-Whitney test. (**B**) Double immunofluorescence for BrdU (red signal) and Ki67 (green signal) shows the lower percentage of double positive proliferating cells (yellow, arrowhead) in Tg2576 neurospheres compared to WT. DAPI staining on nuclei in blue, 40X magnification, zoom 1.25 right panels, scale bars 50 µm and 75 µm, respectively. Quantification is expressed as means ± SEM of five individual animals (n = 5) for each genotype. *P < 0.05, significantly different from WT, Mann-Whitney test. (**C**) No difference in apoptosis between the Tg2576 and WT progenitors. Immunostaining for cleaved caspase-3 (green signal) shows that there is not a significant difference in apoptosis between Tg2576 and WT samples, as quantified in the histogram. DAPI staining on nuclei in blue. Scale bar 50µm, 63X magnification. Data are means ± SEM of two samples for each experimental group examined in three independent experiments (n = 6). n.s., not significantly different from WT, Mann-Whitney test.

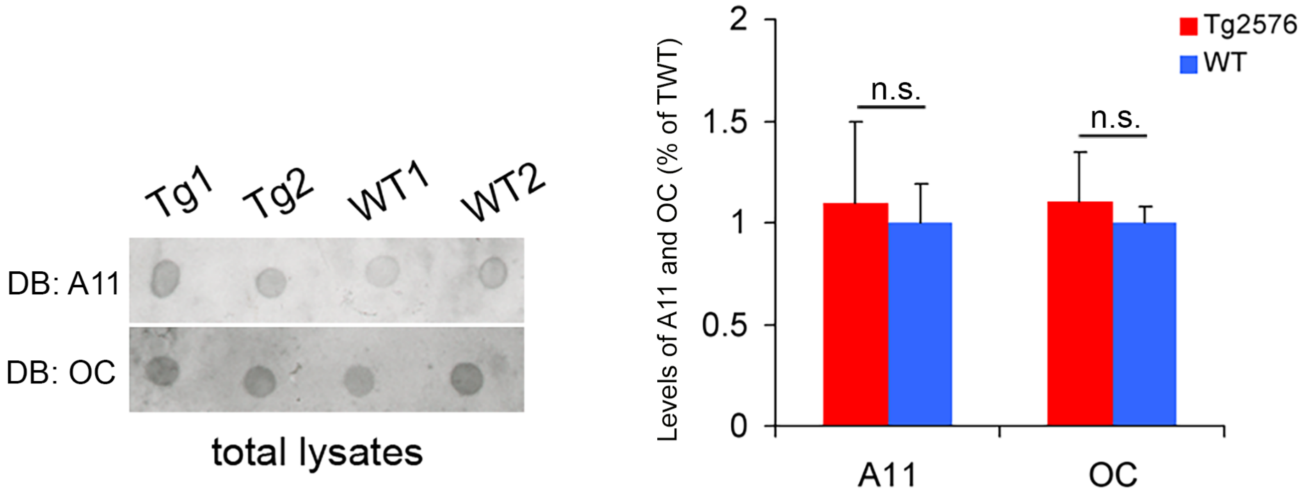

Scopa Supplementary Figure S2

Supplementary Figure S2. Dot blot analysis of Tg2576 and WT total lysates for A11 and OC showing that generic amyloid conformers (such as generic pre-fibrillar and fibrillar oligomers, recognized by A11 and OC antibodies, respectively) do not change. Quantifications are obtained from densitometric values of bands and spots normalized for β-actin. Data are means ± SEM of two samples for each experimental group examined in three independent experiments (n = 6). n.s., not significantly different from WT, Student's t-test.

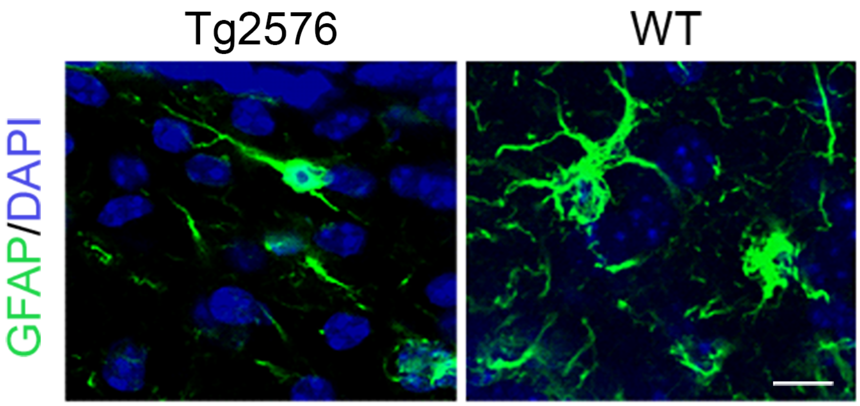

Scopa Supplementary Figure S3

Supplementary Figure S3. Altered astrocytes morphology in Tg2576 SVZ.

Immunofluorescence staining for GFAP (green) in Tg2576 and WT SVZ. Also *in vivo*, Tg2576 astrocytes show an evident altered, elongated cell shape, respect to their control WT counterpart. DAPI staining on nuclei in blue. Scale bar, 10 μm (63X magnification).

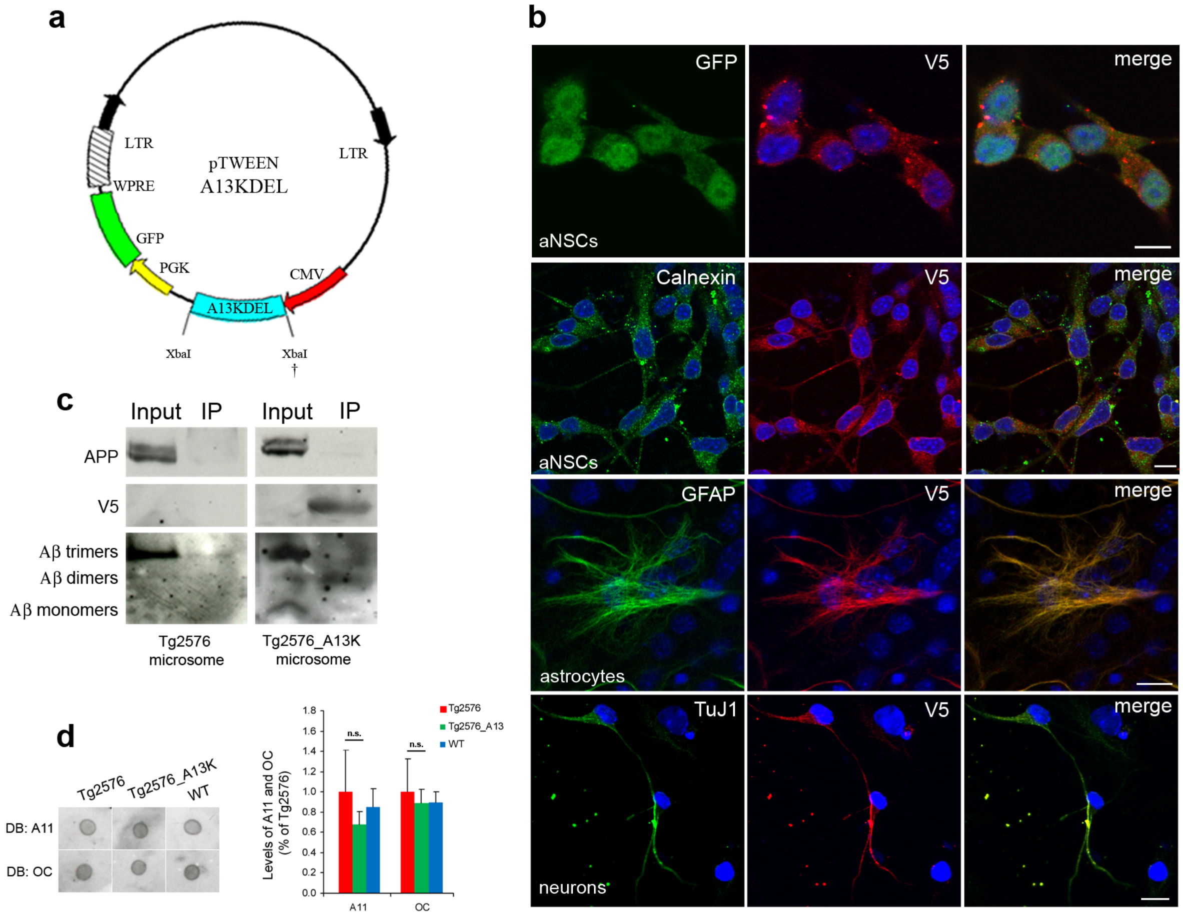

Scopa Supplementary Figure S4

Supplementary Figure S4. Lentiviral delivery of scFvA13-KDEL intrabody in Tg2576 progenitors.

(**A**) Plasmid map of pTWEEN A13K-DEL vector used to generate the lentiviral particles. The bicistronic plasmid, carrying the GFP sequence under the PGK promoter, contains the scFvA13-KDEL cDNA cloned downstream the CMV promoter and upstream the PGK_GFP cassette. (**B**) Tg2576 infected GFP^+ progenitors (Tg2576_A13K, green signal, left panel) stably express scFvA13-KDEL intrabody in undifferentiated progenitors (NSC), as shown by immunofluorescence staining for its C-terminal V5 tag (red and merged signal in central and right panels, respectively). The co-localization of V5 (red) with the ER marker Calnexin (green) demonstrates the ER localization of the intrabody (merged signal in right panel). scFvA13-KDEL expression (in red, central bottom panels) is maintained upon differentiation of the progenitors into $GFAP^+$ astrocytes and $Tuj1^+$ neurons (green and merged signal in left and right bottom panels, respectively). Scale bar 10 μm, 63X magnification, zoom factor from top to bottom panels: 2.5 (GFP/V5 staining), 2 (Calnexin/V5 staining) and 3.5 (TuJ1/V5 and GFAP/V5 staining). (**C**) Intrabody-mediated selective pull-down of AβOs: co-IP of scFvA13-KDEL intrabody mainly with Aβ dimers, but not with APP and Aβ monomer, from microsome of Tg2576_A13K cells and not from Tg2576. The co-IP was performed by using an anti-V5 coupled resin to bind the C-terminal V5 tag of the intrabody, and then blotted with mAb D54D2 and mAb WO2 to detect the different Aβ species and APP, respectively, and with anti-V5 for the intrabody detection. On bottom, the histogram represents the quantification of the representative Aβ and V5 bands detected and normalized for APP. (**D**) Dot blot analysis of Tg2576, Tg2576_A13K and WT total lysates for A11 and OC

demonstrates that generic pre-fibrillar oligomers and fibrillar oligomers remained unchanged. Quantifications are reported in comparison with Tg2576 samples (at 1), obtained from densitometric values of spots normalized for β-actin. Data are means ± SEM of two samples for each experimental group examined in three independent experiments (n = 6). n.s., not significantly different from Tg2576, Student's t-test.

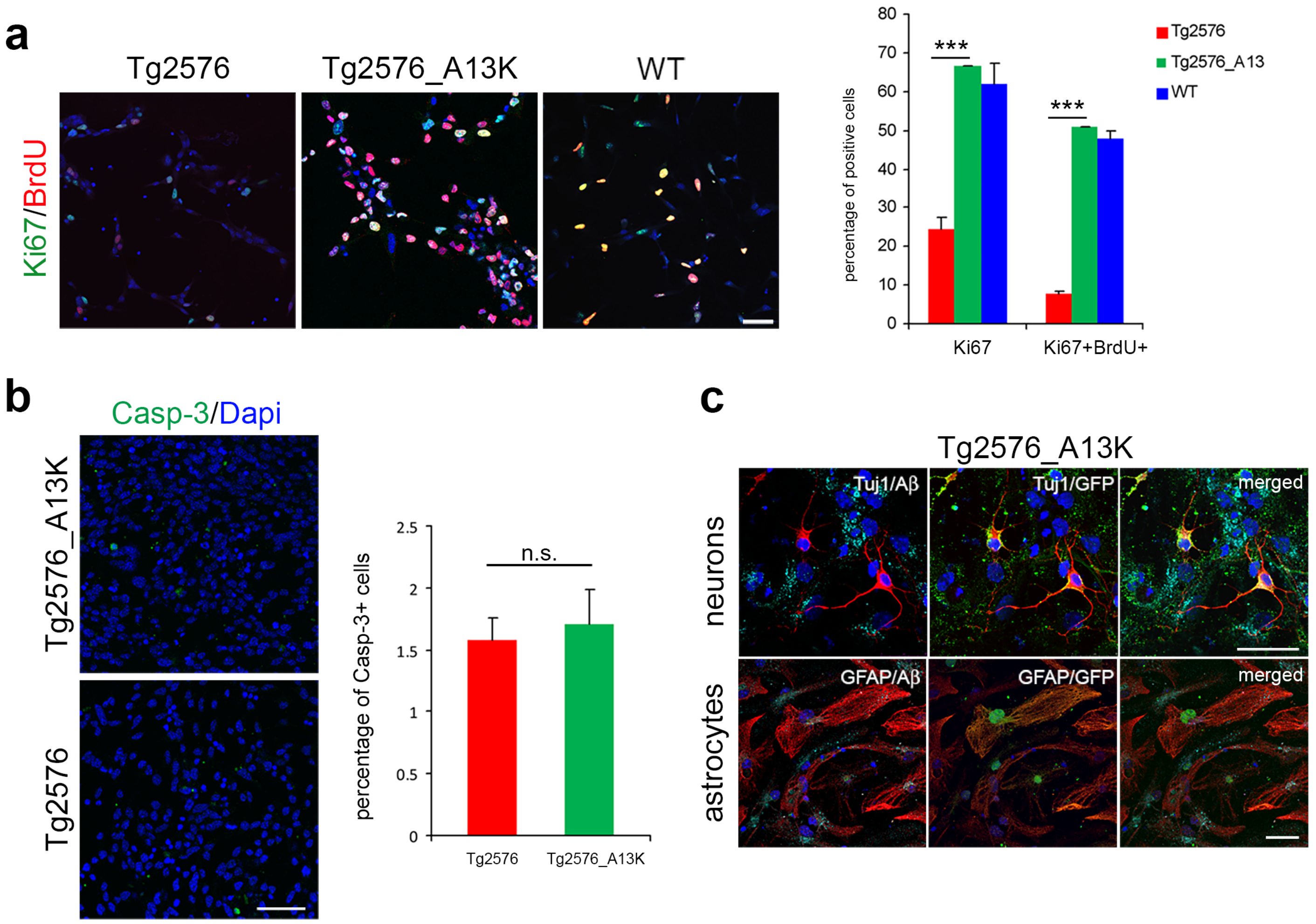

Scopa Supplementary Figure S5

Supplementary Fig. S5. Effect of scFvA13-KDEL intrabody on proliferation and differentiation of Tg2576 aNSCs.

(**A**) Immunostaining for BrdU (red) and Ki67 (green) shows that in Tg2576_A13K samples there are more $BrdU^{+}$ and double $BrdU^{+}Ki67^{+}$ cells, confirming the rescue of proliferation of Tg2576 aNSCs by scFvA13-KDEL. DAPI staining on nuclei in blue. Scale bar 50 µm, 63X magnification. Quantification of percentage of $BrdU^{+}$ cells, $Ki67^{+}$ cells and double positive ($BrdU^{+}Ki67^{+}$) cells in Tg2576, Tg2576_A13K and WT samples is expressed as mean ± SEM of two samples for each experimental group examined in three independent experiments (n = 6). ***$P < 0.001$, significantly different from Tg2576, Mann-Whitney test. (**B**) Similar percentage of apoptotic cells are present in Tg2576 and Tg2576_A13K progenitor populations, as shown by the immunostaining for cleaved activated caspase-3 (green signal) and the quantification in the histogram**.** DAPI staining on nuclei in blue. Scale bar 50 µm, 63X magnification. Data are means ± SEM of two samples for each experimental group examined in three independent experiments (n = 6). n.s., not significantly different from WT, Mann-Whitney test. (**C**) Immunofluorescence staining for Aβ (cyan) and TuJ1 or GFAP (red) in Tg2576_A13K differentiated GFP^{+} cells (green) shows that infected neurons and astrocytes recover their proper cell morphology. Scale bar 25 µm, 63X magnification, zoom 1.3 and 2.5 (neurons and astrocytes panels, respectively).

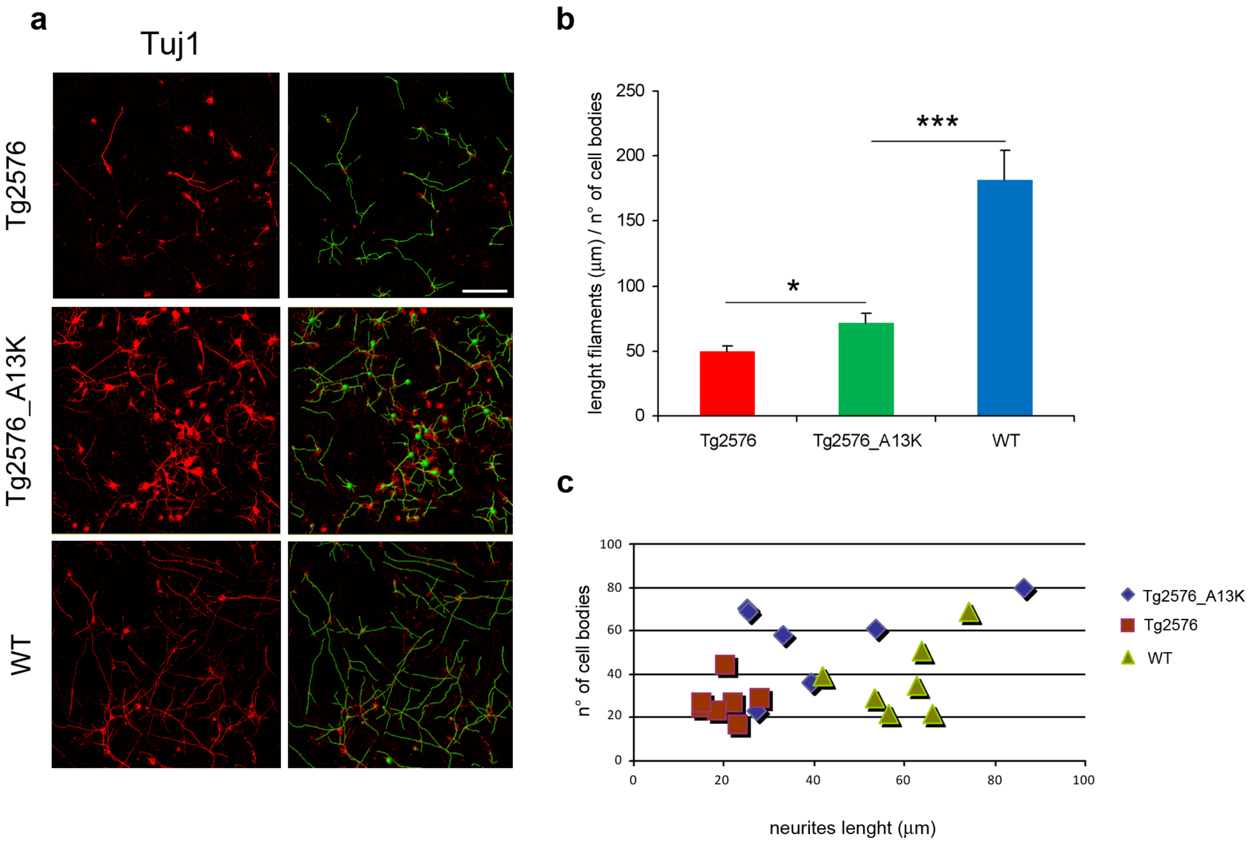

Scopa Supplementary Figure S6

Supplementary Figure 6. Rescue of neuronal differentiation of Tg2576 progenitors by scFvA13-KDEL intrabody expression.

(**A**) Left panels: immunofluorescence staining for neurons (TuJ1, red signal) highlights scFvA13-KDEL intrabody effect on the degree of Tg2576 neuronal maturation. Green signal in right panels reports IMARIS software quantification. In Tg2576_A13K samples, neurites length is partially rescued to that measured in WT sample, as quantified in **B**. Scale bar 25µm, 40X magnification, zoom 1.5. Data are means ± SEM of five individual animals (n = 5) for each experimental group. *P < 0.05, significantly different from Tg2576, and ***P < 0.001, significantly different from WT, Anova test. (**C**) Graph reporting neurites length for each cell body.

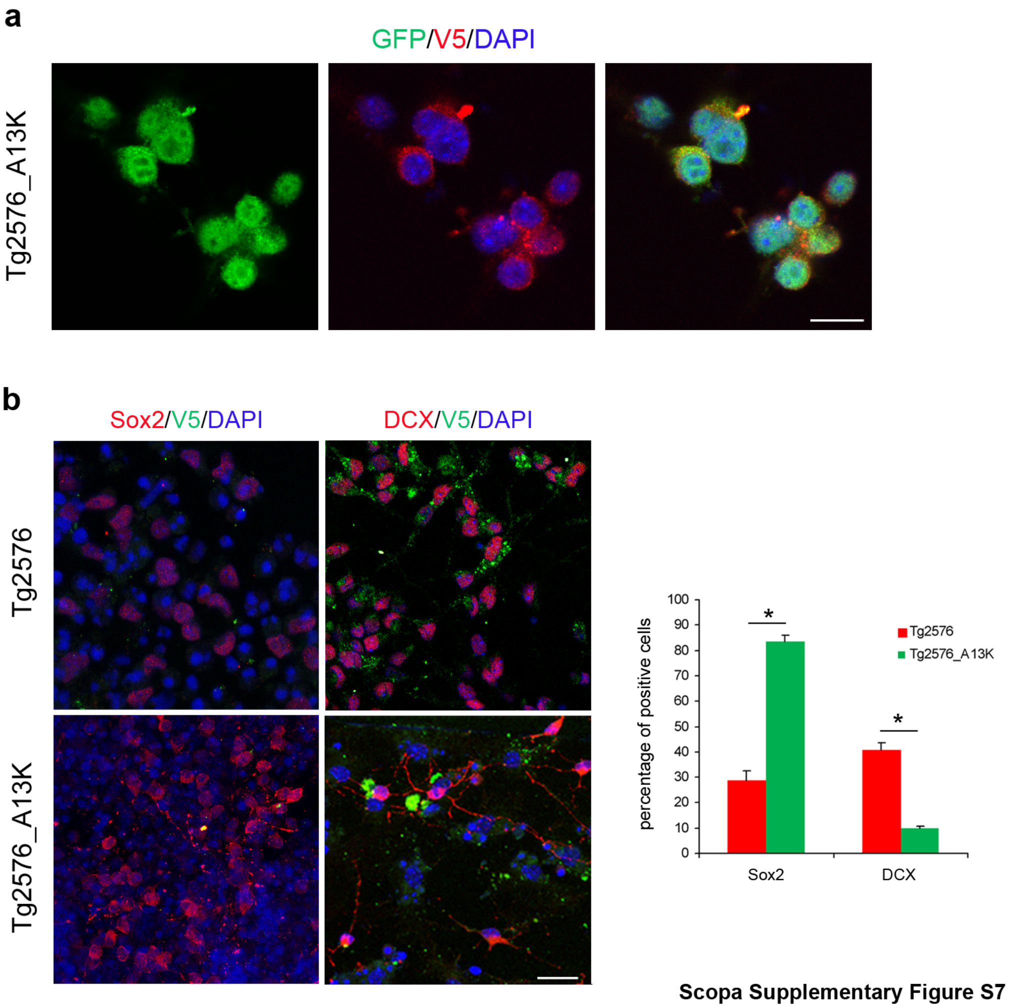

Scopa Supplementary Figure S7

Supplementary Figure S7. *Ex-vivo* rescue of Tg2576 impaired neurogenesis by scFvA13-KDEL lentiviral infection.

(**A**) aNSCs derived from infected SVZ (Tg2576_A13K) express both A13 intrabody (V5, red signal) and GFP (green signal). DAPI staining on nuclei in blue. Scale bar 10 µm, 63X magnification, zoom 2. (**B**) The intrabody expression (V5 staining, green signal) in Sox2 and Dcx cells (red signal in left and in right panels, respectively) restores the correct number of progenitors and neuroblasts in Tg2576_A13K neurospheres culture, as quantified in the histogram. Quantification are expressed as means ± SEM of five individual animals ($n = 5$) for each genotype. *$P < 0.05$, significantly different from WT, Mann-Whitney test.

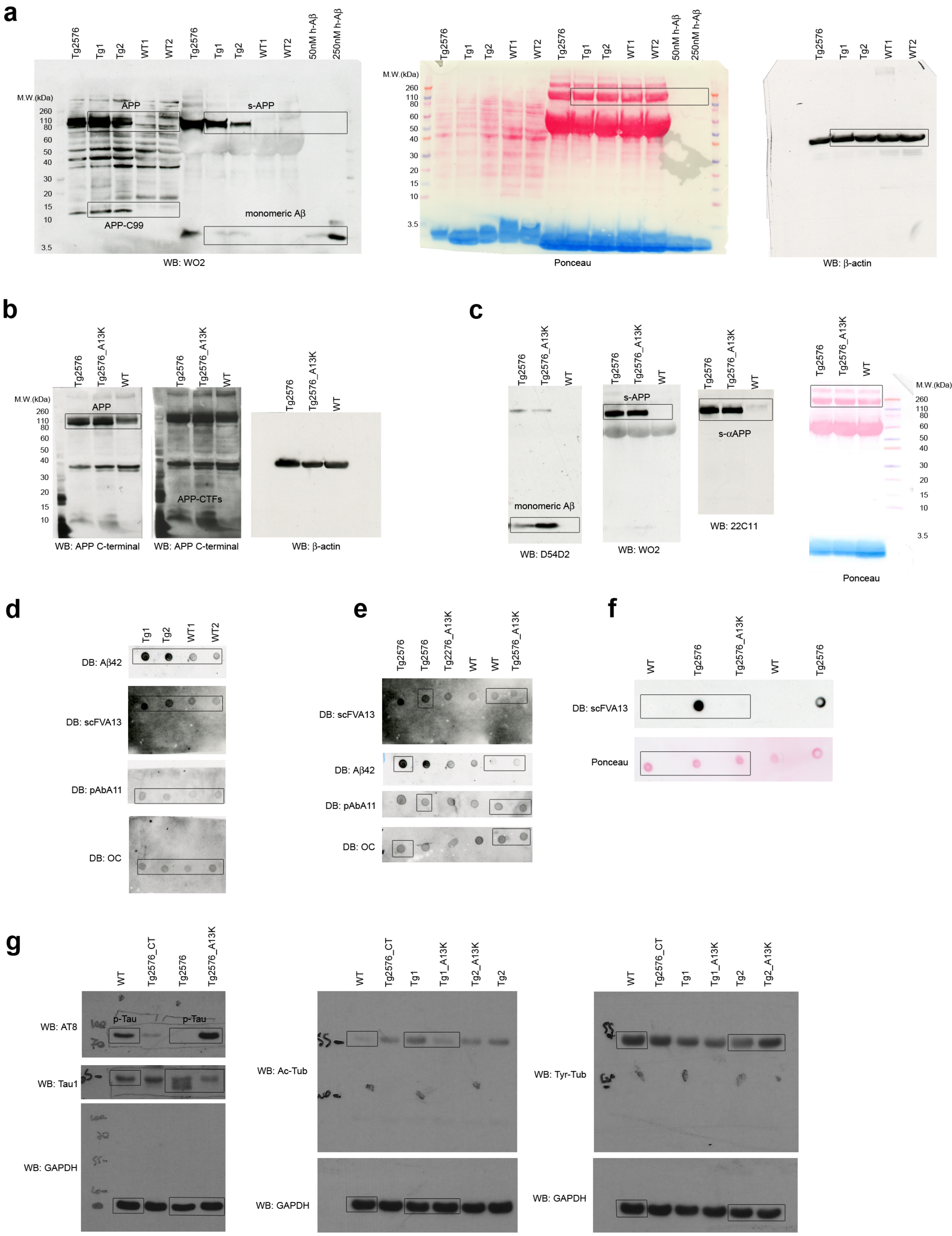

Scopa Supplementary Figure S8

Supplementary Figure S8. **Full-lenght gels of Western blot and and full-length dot blots.**

(**A-C**) Full-length gels of Western blot (WB) analyses of cell lysates and conditioned media (CM), as reported in Figures 2E and 2F (**A**), 4A (**B**) and 4B (**C**). (**D-F**) Full-length of Dot blot (DB) analyses represented in Figure 2E and Supplementary Fig. S2 (**D**), 4C and Supplementary Fig. S4D (**E**) and 4D (**F**). Ponceau Red staining of the nitrocellulose membrane, before the incubation with the different antibodies for WB and DB analyses of CM described above. Similar loads of proteins can be observed, as normalization control. (**G**) Full-length gels of Western blot analyses of cell lysates as reported in Figure 5A. Black squared boxes in each panels indicate the representative lanes or dots chosen to assemble the corresponding main figures.
